# Supplementary material for: Transcellular progression of infection threads in Medicago truncatula roots is associated with locally confined cell wall modifications
Source: Curr Biol. 2023 Feb 6;33(3):533–542.e5. doi: 10.1016/j.cub.2022.12.051 (PMC9937034; doi:10.1016/j.cub.2022.12.051)
Supplement: Document S1. Figures S1–S4 [file mmc1.pdf]

Current Biology, Volume *33*

Supplemental Information

Transcellular progression of infection threads in *Medicago truncatula* roots is associated with locally confined cell wall modifications

Chao Su, Guofeng Zhang, Marta Rodriguez-Franco, Rosula Hinnenberg, Jenny Wietschorke, Pengbo Liang, Wei Yang, Leonard Uhler, Xia Li, and Thomas Ott

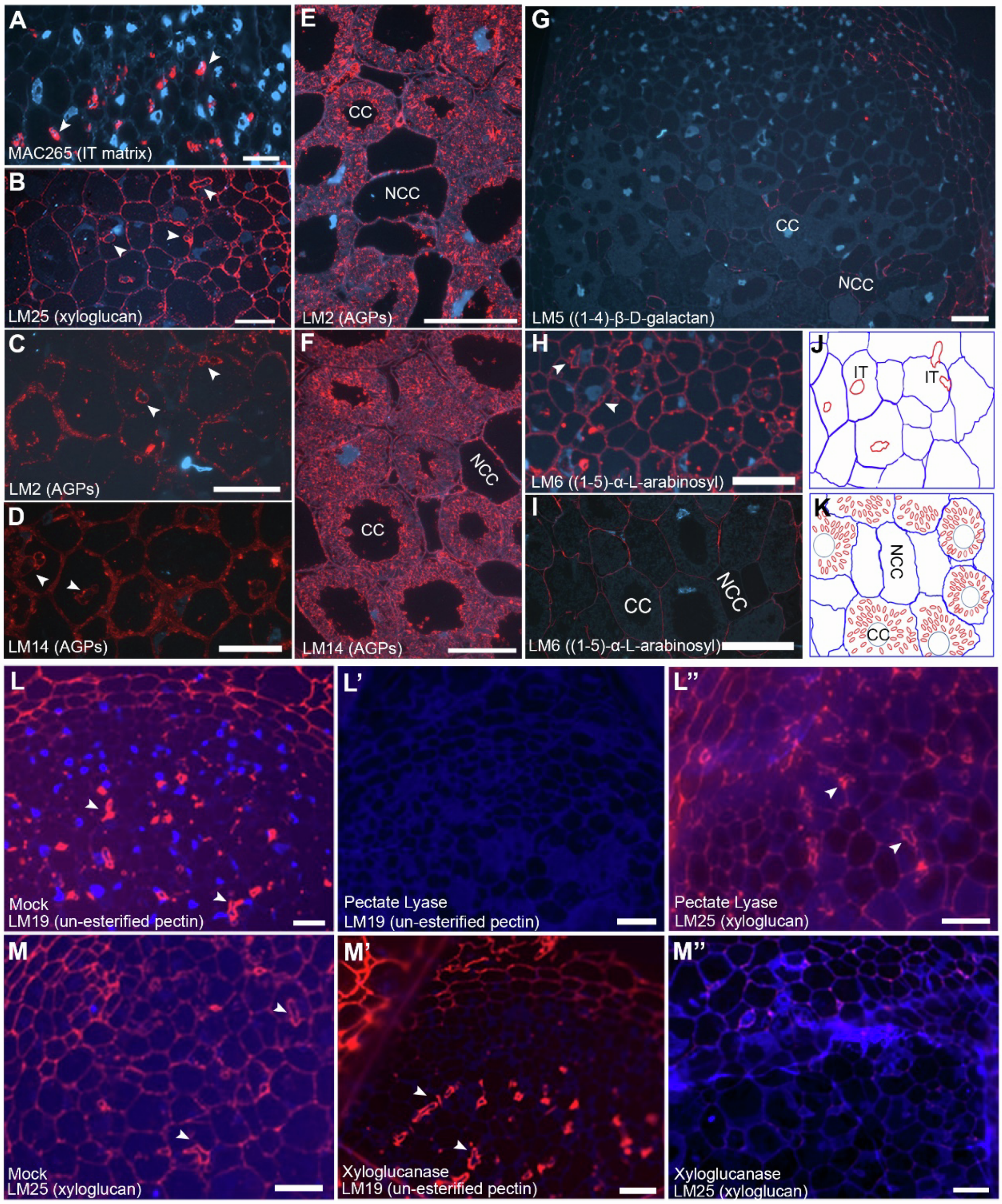

Figure S1: Immunofluorescence labeling with different cell wall antibodies, related to Figure 1. (A-I) 14 days old Medicago nodules were embedded in Lowicryl HM20 and hybridized with the respective antibodies (red). Cellular structures were labelled by different antibodies: (A) Infection thread (IT) matrix (MAC265); (B) xyloglucan (LM25); (C-F) arabinogalactan proteins (AGPs; LM2 (C, E), LM14 (D, F); different types of RG-I (LM5 (G) and LM6 (H, I)). Sections of nodule infection zone (A-D, H) and fixation zone (E, F, and I). (J-K) Illustrations indicate nodule sections from infection zone (J) and fixation zone (K). Red circles in (J) indicate ITs and symbiosomes in (K). Images in B, C, D and H as well as E and F were taken from consecutive sections from the same nodules but probed with different antibodies, respectively. (L-M”). Nodule sections were pre-treated with pectate lyase (L-L”) or xyloglucanase (M-M”) before being hybridized with different antibodies. DNA was counterstained with DAPI (blue). Images in L-M” were taken from consecutive sections from the same nodule but probed with different antibodies. Arrowheads indicate infection threads. Scale bars indicate 50 µm. IT: infection thread, CC: colonized cell; NCC: non-colonized cell.

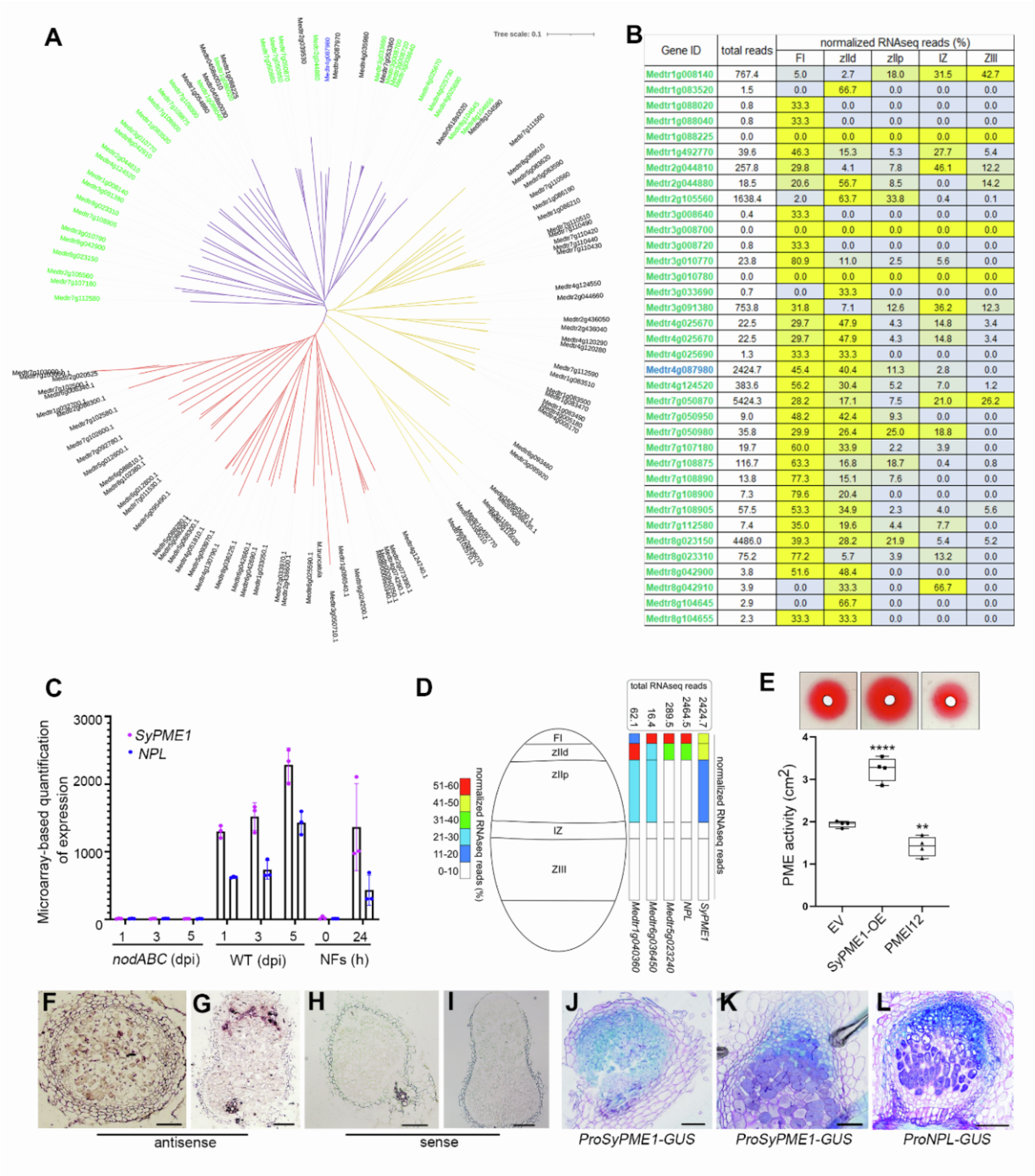

Gene ID	total reads	normalized RNAseq reads (%)				
		FI	zIId	zIIp	IZ	ZIII
Medtr1g008140	767.4	5.0	2.7	18.0	31.5	42.7
Medtr1g083520	1.5	0.0	66.7	0.0	0.0	0.0
Medtr1g088020	0.8	33.3	0.0	0.0	0.0	0.0
Medtr1g088040	0.8	33.3	0.0	0.0	0.0	0.0
Medtr1g088225	0.0	0.0	0.0	0.0	0.0	0.0
Medtr1g492770	39.6	46.3	15.3	5.3	27.7	5.4
Medtr2g044810	257.8	29.8	4.1	7.8	46.1	12.2
Medtr2g044880	18.5	20.6	56.7	8.5	0.0	14.2
Medtr2g105560	1638.4	2.0	63.7	33.8	0.4	0.1
Medtr3g008640	0.4	33.3	0.0	0.0	0.0	0.0
Medtr3g008700	0.0	0.0	0.0	0.0	0.0	0.0
Medtr3g008720	0.8	33.3	0.0	0.0	0.0	0.0
Medtr3g010770	23.8	80.9	11.0	2.5	5.6	0.0
Medtr3g010780	0.0	0.0	0.0	0.0	0.0	0.0
Medtr3g033690	0.7	0.0	33.3	0.0	0.0	0.0
Medtr3g091380	753.8	31.8	7.1	12.6	36.2	12.3
Medtr4g025670	22.5	29.7	47.9	4.3	14.8	3.4
Medtr4g025670	22.5	29.7	47.9	4.3	14.8	3.4
Medtr4g025690	1.3	33.3	33.3	0.0	0.0	0.0
Medtr4g087980	2424.7	45.4	40.4	11.3	2.8	0.0
Medtr4g124520	383.6	56.2	30.4	5.2	7.0	1.2
Medtr7g050870	5424.3	28.2	17.1	7.5	21.0	26.2
Medtr7g050950	9.0	48.2	42.4	9.3	0.0	0.0
Medtr7g050980	35.8	29.9	26.4	25.0	18.8	0.0
Medtr7g107180	19.7	60.0	33.9	2.2	3.9	0.0
Medtr7g108875	116.7	63.3	16.8	18.7	0.4	0.8
Medtr7g108890	13.8	77.3	15.1	7.6	0.0	0.0
Medtr7g108900	7.3	79.6	20.4	0.0	0.0	0.0
Medtr7g108905	57.5	53.3	34.9	2.3	4.0	5.6
Medtr7g112580	7.4	35.0	19.6	4.4	7.7	0.0
Medtr8g023150	4486.0	39.3	28.2	21.9	5.4	5.2
Medtr8g023310	75.2	77.2	5.7	3.9	13.2	0.0
Medtr8g042900	3.8	51.6	48.4	0.0	0.0	0.0
Medtr8g042910	3.9	0.0	33.3	0.0	66.7	0.0
Medtr8g104645	2.9	0.0	66.7	0.0	0.0	0.0
Medtr8g104655	2.3	33.3	33.3	0.0	0.0	0.0

Figure S2: Analysis of *SyPME* expression domains, related to Figures 2 and 3. (A) Maximum likelihood tree of the PME family in Medicago. All the (amino acid) sequences were obtained from *Phytozome v13* (https://phytozome-next.jgi.doe.gov/info/Mtruncatula_Mt4_0v1). The gene marked in blue is *SyPME1*. Genes in green letters fall into SyPME1 clade (A) and gene expression data for these genes were retrieved from [S1] (B). FI: nodule meristematic zone; zIId: distal of infection zone; zIIp: proximal of infection zone; IZ: interzone; ZIII: nitrogen-fixation zone. (C) Expression patterns of *NPL* and *SyPME1* are highly correlated (0.9883) as exemplified upon inoculation of roots with a Nod Factor (NF)-deficient *S. meliloti nodABC* strain (*nodABC*), WT *S. meliloti* and isolated NFs at different time points. Data were retrieved from [S2]. dpi: days post inoculation; h: hours. (D) Schematic overview of the spatial expression of SyPME and different pectate lyases including NPL in different zones of an indeterminate Medicago nodule. Original data were retrieved from [S1]. (E) Quantification analysis of PME activity from inoculated roots containing empty vector (EV), SyPME1-GFP (SyPME-OE), and PMEI12-GFP (PMEI12) (n = 4). Data are means ± SE. Statistics were performed using an unpaired two-tailed t-test: ****p < 0.0001; **p < 0.01. (F-I) Spatial analysis of *SyPME* transcript accumulations by i*n situ* hybridization using a *SyPME* antisense (F-G) and sense (control) probe (H-I) on 14-day old Medicago nodules. Magenta precipitates indicate presence of *SyPME* mRNA. (J-L) Promoter GUS (blue) analysis for *ProSyPME1* (J, K) and *ProNPL* (L) on 14-day old transformed *M. truncatula* nodules counterstained with Toluidine Blue (purple). Scale bars indicate 50 µm.

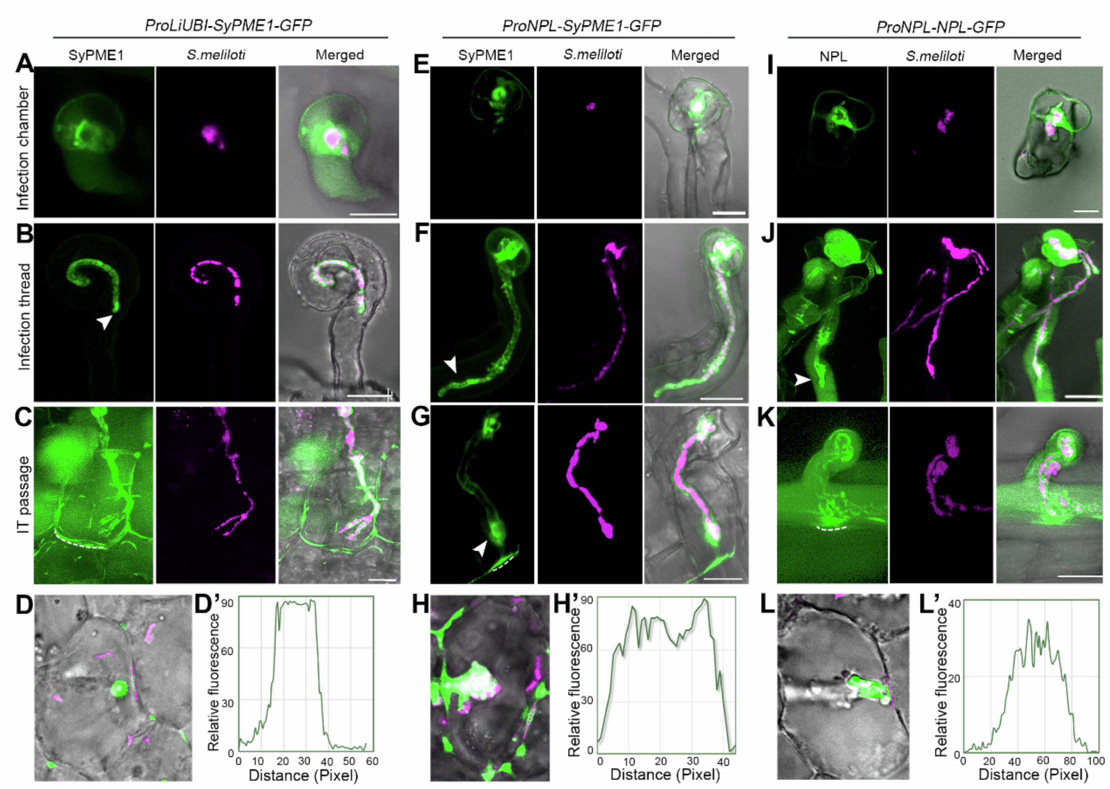

Figure S3: SyPME1 and NPL accumulate in spatially overlapping sites, related to Figure 2. Expression of a SyPME1-GFP fusion protein (green) was driven by the *ProLjUBI* promoter (A-D) or the *ProNPL* promoter (E-H). Expression of an NPL-GFP fusion protein (green) was driven by *ProNPL* (I-L). (A-B, E-F) SyPME1-GFP (green) localizes to the infection chamber (IC; A, E (n = 20)) and along growing infection threads (IT) including the IT tip (arrowhead, B, F (n = 15)). (I-J) Similar patterns were observed for NPL-GFP (green) at the IC (I, n = 20) while NPL predominantly localized to the IT tip (arrowhead) and young parts of the IT (J, n = 20). Initial labelling of future transcellular passage sites prior to IT passage was found for SyPME1 (C, G) and NPL (K) as indicated by the dashed line (n = 5 for C, n = 6 for G and n = 4 for K). Fluorescent *S. meliloti* is shown in magenta. All images are maximal projections. Scale bars indicate 10 µm. Inside nodule, SyPME1 (D, H) and NPL (L) also accumulate at prospective passage sites and at the tip of IT (images are identical with panels Figure 3B, H, N, respectively). Dashed magenta line indicates transect used to determine fluorescence intensities at these sites for SyPME1 (D', H') and NPL (L'). Data were collected based on at least three independent rounds of hairy root transformations.

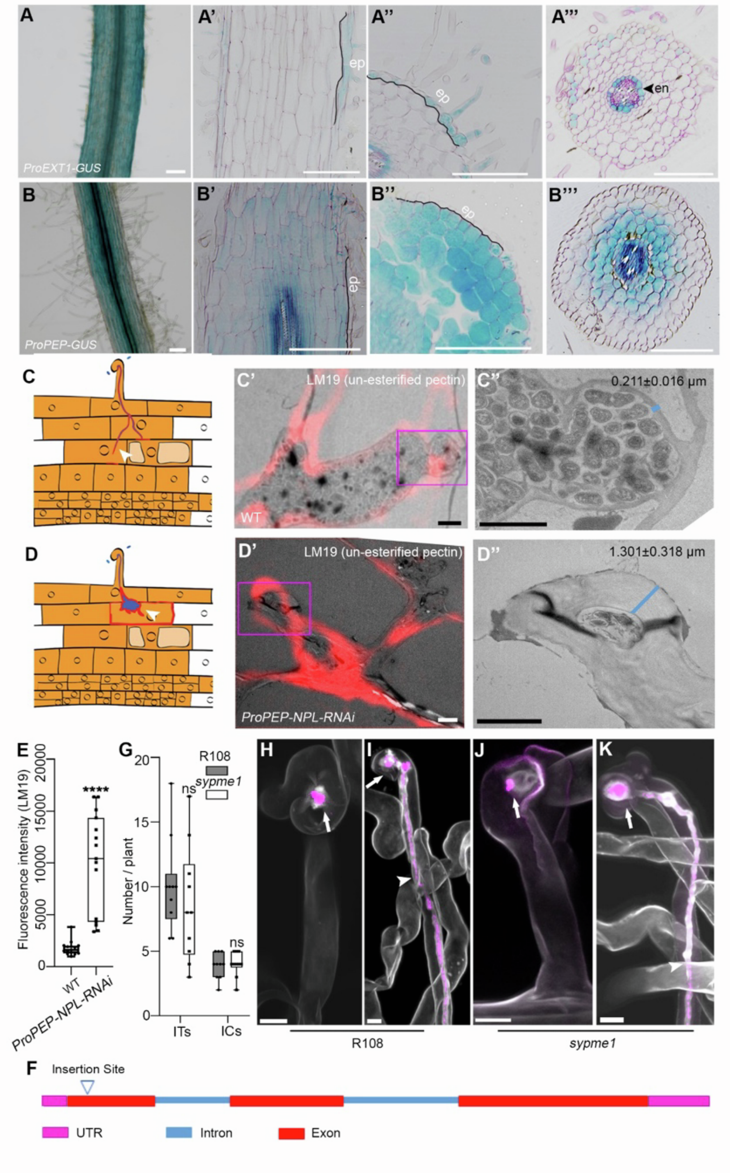

Figure S4: A single locus *sypme1* mutant does not exhibit a symbiotic phenotype, related to Figures 3 and 4. (A-B''') GUS staining of Medicago roots transformed with *ProEXT1-GUS* (A-A''') and *ProPEP-GUS* (B-B''') reporter constructs. Root longitudinal sections (A' and B') and cross sections (A''-A''', B''-B''') after GUS staining. ep: epidermis, en: endodermis. Scale bars indicate 100 µm. (C and D) Models illustrating the sites (arrowheads) that were used for CLEM analysis. (C-D'') CLEM analysis of sections of cortical cells from WT (C-C'', n=4) and *ProPEP-NPL-RNAi* transgenic roots (D-D'', n=3) penetrated by ITs and labelled with LM19 (red, C' and D'). (C'' and D'') Close-up of the boxed region (magenta) in C' and D', respectively. The blue bars in C'' and D'' indicate the thickness of IT cell walls and the numbers indicate means of 15 measurements from 3 independent samples. Scale bars indicate 2 µm. Quantitative immune-labelling of un-esterified pectins in (C' and D') is shown in E (n=15). Data are means ± SE. Statistics were performed using an unpaired two-tailed t-test: ****$p < 0.0001$. (F) Schematic representation of the *SyPME1* gene structure and mapped Tnt1 transposon insertion site (line NF2281). UTR, untranslated region. (G) Infection chambers (ICs) and infection threads (ITs) were scored at 10 days after inoculation, with n =10 root systems for each genotype. Data are means ± SE. Statistics were performed using an unpaired two-tailed t-test. ns: Not significant. (H-K) IC and IT morphology were visualized by Calcofluor-white staining (White color) in R108 (H-I) and *sypme1* mutants (J-K). Fluorescent *S. meliloti* is shown in magenta. Arrows indicate the IC and arrowheads indicate the IT. Scale bars indicate 10 µm. Images (H-K) are maximal projections.

SUPPLEMENTAL REFERENCES

S1. Roux, B., Rodde, N., Jardinaud, M.F., Timmers, T., Sauviac, L., Cottret, L., Carrere, S., Sallet, E., Courcelle, E., Moreau, S., et al. (2014). An integrated analysis of plant and bacterial gene expression in symbiotic root nodules using laser-capture microdissection coupled to RNA sequencing. Plant J *77*, 817-837. 10.1111/tpj.12442.

S2. Breakspear, A., Liu, C., Roy, S., Stacey, N., Rogers, C., Trick, M., Morieri, G., Mysore, K.S., Wen, J., Oldroyd, G.E., et al. (2014). The root hair "infectome" of Medicago truncatula uncovers changes in cell cycle genes and reveals a requirement for Auxin signaling in rhizobial infection. Plant Cell *26*, 4680-4701. 10.1105/tpc.114.133496.
