# Supplementary material for: Transcellular progression of infection threads in Medicago truncatula roots is associated with locally confined cell wall modifications
Source: Curr Biol. 2023 Feb 6;33(3):533–542.e5. doi: 10.1016/j.cub.2022.12.051 (PMC9937034; doi:10.1016/j.cub.2022.12.051)
Supplement: Document S2. Article plus supplemental information [file mmc2.pdf]

Current Biology

Article

Transcellular progression of infection threads in *Medicago truncatula* roots is associated with locally confined cell wall modifications

Graphical abstract

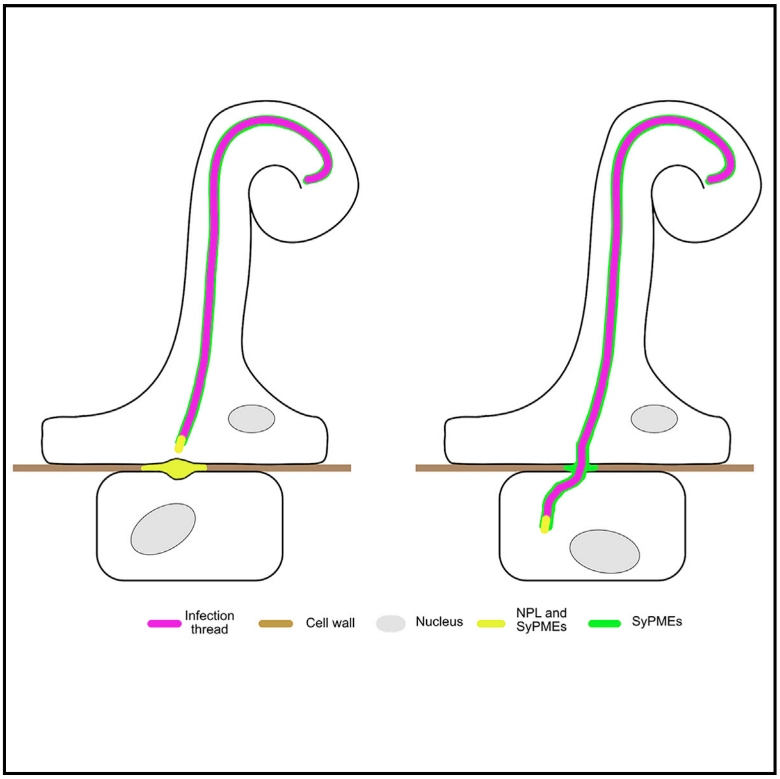

Authors

Chao Su, Guofeng Zhang, Marta Rodriguez-Franco, ..., Leonard Uhler, Xia Li, Thomas Ott

Correspondence

chao.su@biologie.uni-freiburg.de (C.S.), thomas.ott@biologie.uni-freiburg.de (T.O.)

In brief

In this paper, Su et al. demonstrate how the cell wall is dynamically modulated during the transcellular passage of symbiotic infection threads. Using genetics and correlative-light electron microscopy, they unraveled that two enzymes mediate confined pectin modifications that enable this crucial process in *Medicago truncatula* roots and nodules.

Highlights

- NPL is required for transcellular infection thread (IT) passage
- Inhibition of SyPMEs activity affects ITs growth
- NPL and SyPME1 specifically accumulate at symbiotically active sites
- Spatially controlled cell wall modifications are required for IT progression

Su et al., 2023, Current Biology *33*, 533–542
February 6, 2023 © 2022 The Author(s). Published by Elsevier Inc.
https://doi.org/10.1016/j.cub.2022.12.051

Current Biology

CellPress
OPEN ACCESS

Article

Transcellular progression of infection threads in *Medicago truncatula* roots is associated with locally confined cell wall modifications

Chao Su,[1,*] Guofeng Zhang,[1] Marta Rodriguez-Franco,[1] Rosula Hinnenberg,[1] Jenny Wietschorke,[1] Pengbo Liang,[1,2] Wei Yang,[3] Leonard Uhler,[1] Xia Li,[3] and Thomas Ott[1,4,5,6,*]

[1]Cell Biology, Faculty of Biology, University of Freiburg, 79104 Freiburg, Germany
[2]State Key Laboratory of Plant Physiology and Biochemistry, MOA Key Laboratory of Soil Microbiology, and Rhizobium Research Center, College of Biological Sciences, China Agricultural University, Beijing 100193, P.R. China
[3]National Key Laboratory of Crop Genetic Improvement, Hubei Hongshan Laboratory, College of Plant Science and Technology, Huazhong Agricultural University, Hongshan District, Wuhan 430070, Hubei, P.R. China
[4]CIBSS – Centre of Integrative Biological Signalling Studies, University of Freiburg, 79104 Freiburg, Germany
[5]Twitter: @OttLab_Freiburg
[6]Lead contact
*Correspondence: chao.su@biologie.uni-freiburg.de (C.S.), thomas.ott@biologie.uni-freiburg.de (T.O.)
https://doi.org/10.1016/j.cub.2022.12.051

SUMMARY

The root nodule symbiosis with its global impact on nitrogen fertilization of soils is characterized by an intracellular colonization of legume roots by rhizobia. Although the symbionts are initially taken up by morphologically adapted root hairs, rhizobia persistently progress within a membrane-confined infection thread through several root cortical and later nodular cell layers. Throughout this transcellular passaging, rhizobia have to repeatedly pass host plasma membranes and cell walls. Here, we investigated this essential process and describe the concerted action of one of the symbiosis-specific pectin methyl esterases (SyPME1) and the nodulation pectate lyase (NPL) at the infection thread and transcellular passage sites. Their coordinated function mediates spatially confined pectin alterations in the cell-cell interface that result in the establishment of an apoplastic compartment where bacteria are temporarily released into and taken up from the subjacent cell. This process allows successful intracellular progression of infection threads through the entire root cortical tissue.

INTRODUCTION

Legumes evolutionarily maintained the intriguing ability to intracellularly accommodate symbiotic bacteria called “rhizobia.” This mutualistic interaction results in the development of the root nodule symbiosis (RNS) that is morphologically initiated by a re-orientation of root hair (RH) growth, a process named “root hair curling,” followed by bacterial capture within a newly formed structure called the infection chamber (IC).[1] The entrapped rhizobia start dividing inside the IC and trigger the invasive growth of a tunnel-like structure, the infection thread (IT).[2,3] ITs are guided by pre-formed cytosolic columns (pre-ITs) that are rich in endoplasmic reticulum and cytoskeleton components[3,4] toward the basal membrane of the cell. In the course of IT progression, an organogenesis program is executed in root cortical and pericycle cells that results in the development of a nodule primordium.[5,6] ITs will transcellularly grow toward this primordium by penetrating several root cortical cell layers and finally release bacteria to cells inside the nodule. These colonized nodule cells provide the differentiated rhizobia (bacteroids) with an environment that is low in free oxygen and thus enables the fixation of atmospheric dinitrogen gas by the rhizobial nitrogenase complex.[7]

It can be assumed that spatiotemporally confined cell wall (CW) remodeling is required to initiate and maintain IT growth, the transcellular passage of ITs, as well as bacterial release. CWs of dicotyledonous plants mainly consist of cellulose, hemicellulose, pectins, and structural glycoproteins.[8] The polysaccharide callose is deposited in a more locally and temporarily confined manner and the loss of the callose degrading enzyme MtBG2 leads to defects in nodulation.[4]

Homogalacturonan (HG) is the major type of pectin in primary CWs and the middle lamella and is thus found predominantly in young plant tissues. These heteropolysaccharides are synthesized in the Golgi apparatus and transported as methyl-esterified pectins to the plasma membrane where they are secreted into the apoplast.[5] Consequently, the methyl-esterified form of pectins serves as an abundant CW component of most root cell types. Immunolabeling of CW components in legumes revealed that de-esterified/un-esterified pectins delineate Its,[9–12] whereas the IT matrix may predominantly be comprised of hydroxyproline-rich glycoproteins (HRGPs, extensins).[6–8] Although methyl-esterified pectins form a rather soft and gel-like matrix, de-esterification of pectins by pectin-methylesterases (PMEs) enables complexation by calcium ions into a

This is an open access article under the CC BY license (http://creativecommons.org/licenses/by/4.0/).

mostly more rigid state called “egg-box dimer.” As PMEs form rather large and functionally redundant gene families, PME function has been assessed by using genetically encoded PME inhibitors (PMEIs) that simultaneously block multiple PMEs at the site of PMEI accumulation.[13] De-esterified pectins can also be targeted for degradation by pectate lyases.[13] During RNS, a NODULATION PECTATE LYASE (NPL), which is secreted via the VAMP721 pathway, regulates the stiffness of the CW by mediating the degradation of de-esterified pectins.[9,14] As a consequence, most rhizobial infections are arrested at the IC stage in RHs of *npl* mutant plants,[10,14] indicating the importance of CW modifications for the infection process.

Here, we asked how CW modifications and IT transcellular passage through multiple cell layers are coordinated. To address this question, we used genetic and cell biological approaches to decipher CW dynamics and demonstrated that symbiotic PMEs (SyPMEs) function upstream of NPL activity. The concerted action of both enzymes and their differential and locally confined presence can explain the transcellular passage of ITs during symbiotic interactions.

RESULTS

Analysis of cell wall structures surrounding ITs

To study CW structure and composition during rhizobial infections and transcellular IT passage, we ran a set of pilot immuno-labeling experiments on indeterminate *Medicago truncatula* (hereafter Medicago) nodules using a series of selected antibodies against different CW constituents (key resources table). To identify ITs, we first labeled IT matrix glycoproteins using the MAC265 antibody.[6] As expected, nodular ITs were specifically labeled by this approach, whereas the peripheral CW of nodule cortex cells did not show any fluorescent signal (Figure S1A). This was different when targeting xyloglucan as the most abundant hemicellulose by the LM25 antibody. Here, we found ubiquitous labeling of the cell periphery of all nodule cells including ITs (Figure S1B). In contrast, different arabinogalactan proteins (AGPs, labeled by LM2 and LM14) that have been reported to serve functions during plant-microbe interactions[11] were found to specifically accumulate within the infection zone (Figures S1C, S1D, and S1J) and around symbiosomes (Figures S1E, S1F, and S1K). Next, we addressed the presence of different pectins in nodular tissues. The linear (1-4)-β-D-galactan (recognized by LM5), an epitope of the pectin subtype rhamnogalacturonan I (RG-I), was barely detectable inside Medicago nodule sections (Figure S1G). This is consistent with previously published data, where this epitope was almost absent in nodule sections from Medicago.[7] By contrast, (1-5)-α-L-arabinosyl, another epitope of RG-I that is recognized by LM6, is present in most CWs of cells within the infection zone of the nodule (Figure S1H) and predominantly accumulates in the periphery of colonized cells of the fixation zone (Figure S1I), whereas uninfected cells within this zone did not accumulate (1-5)-α-L-arabinosyl (Figure S1I). To differentiate between HG subtypes, we applied two antibodies, LM20 and LM19, recognizing methyl-esterified and un-esterified HGs, respectively. Although esterified pectins were present in most root CWs (Figure 1A), un-esterified pectins predominantly accumulated in epidermal cells, outer cortical cells, and around ITs, whereas the CW of uninfected and infected cells of the inner nodule cortex were devoid of this processed form of pectin (Figure 1B). Furthermore, and as shown for nodular tissue, un-esterified (Figure 1D) but not methyl-esterified pectins (Figure 1C) accumulated around ITs in nodule cells. We further noticed that labeling of un-esterified pectins frequently extended slightly from the ITs toward neighboring cells (Figures 1E–1E″).

Next, we addressed, whether the specific distribution pattern of un-esterified HG (labeled by LM19) that we observed was influenced by masking effects of other CW components such as xyloglucans. A first, control experiments showed that almost all LM19 antibody signal was lost when treating nodule sections with pectate lyase prior to immuno-labeling, whereas signals for xyloglucan labeled by LM25 antibodies were retained (Figures S1L–S1L″). Next, we incubated nodule sections with xyloglucanase prior to immuno-labeling of un-esterified HGs. Although immuno-labeling of xyloglucans (Figure S1M) and un-esterified pectins (Figure S1M′) were clearly detectable in mock and xyloglucanase-treated samples, respectively, no xylogucans were detectable after xylogucanase treatment anymore (Figure S1M″). Since pectin labeling was unaffected in both untreated and xyloglucanase-treated nodule sections (Figures S1M–S1M″), we concluded that the observed patterns are not influenced by epitope masking of xyloglucans.

Taken together, we demonstrated that the detection of pectin derivatives provides a versatile tool to monitor CW compositions along nodular ITs and around transcellular passage sites.

Assessing the ultrastructure of ITs using correlative-light electron microscopy

In order to dissect CW patterns at transcellular IT passage sites with ultrastructural resolution, we established a correlative-light electron microscopy (CLEM) protocol that allows searching for events by fluorescence microscopy (Figures 1E and 1F) and later perfectly retrieving these sites in ultrathin sections using transmission electron microscopy (TEM) (Figures 1E′ and 1F′). Overlaying those images revealed that un-esterified pectins were present along the ITs and small segments of the host CW being in close proximity to the IT (Figures 1E″ and F″). These sites could be transcellular passage sites as frequently seen using scanning electron microscopy (SEM) (Figure 1G) and TEM (Figure 1H). Those observations were further confirmed by double immuno-gold labeling, which showed un-esterified pectins (LM19, 12 nm) being concentrated at the IT penetration site, whereas only a few gold particles were detected using LM20 (methyl-esterified pectins, 5 nm) (Figures 1I and 1J). These images also revealed that CW structures at transcellular IT passage sites are fused and thickened (Figure 1H). To assess whether this was a result of CW loosening and subsequent swelling or rather represents rigidified structures, we probed these samples using the 2F4 antibody, which recognizes egg-box pectin dimers. Indeed, 2F4 immunofluorescence staining confirmed the accumulation of Ca^{2+} -complexed pectin around ITs and at the transcellular passage sites (Figure 1K). This is in agreement with the observed enrichment of un-esterified pectins around ITs (Figures 1B, 1D, and 1E–1E″), as Ca^{2+}-complexation requires de-methylesterification of HGs.

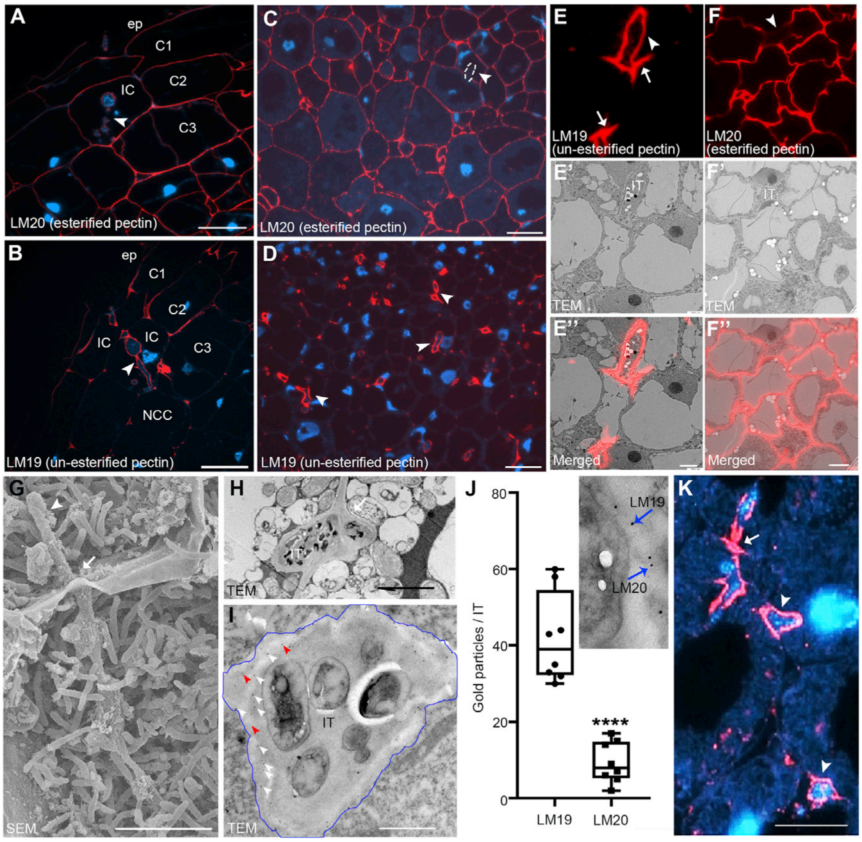

Figure 1. Infection-related modifications of pectins

(A–D) Esterified pectins (LM20, red) are present on the walls of root cortical cells (A) and all cell types in 14-day-old Medicago nodules (C). They only weakly accumulate on the infection thread (IT; arrowheads and indicated by the white dashed line). Enrichment of un-esterified pectins (LM19, red) in infection threads in root cortical cells (B; arrowhead) and peripheral nodule cells with strong accumulations at nodular infection threads (D; arrowheads). Images in (A) and (B) were taken from consecutive sections from the same nodule but probed with two different antibodies. DNA was counterstained with DAPI (blue); ep, epidermis; C1, 1st cortical cell layer; C2, 2nd cortical cell layer; C3, 3rd cortical cell layer; IC, infected cell; NCC, non-colonized cell.

(E–F″) CLEM analysis of esterified (E)–(E″) and un-esterified (F)–(F″) pectins showing the fluorescence image after immuno-labeling of ultrathin sections (E) and (F), the corresponding TEM image (E′) and (F′) and the corresponding merged images (E″) and (F″). Nodular infection threads (IT) are indicated by arrowheads (E) and (F) and extensions of un-esterified pectin toward the direct neighboring cell at potential transcellular passage sites are labeled by arrows (E).

(G and H) Transcellular passage site (arrows) with a nodular infection thread (IT; arrowhead) passing from cell to cell as shown by scanning electron microscopy image (SEM) (G) and transmission electron microscopy (TEM) (H).

(I) Double immuno-gold labeling of un-esterified (LM19; 12 nm) and esterified (LM20; 5 nm) pectins at a nodular infection thread passage site showed labeling of the IT periphery. The IT periphery is encircled by a blue circle line; white and red arrowheads indicate immuno-gold labeled by LM19 and LM20, respectively.

(J) Quantification data for double immuno-gold labeling using LM19/LM20. Gold particles with different grain sizes were counted per IT (n = 8). Data are mean ± SE. Statistics were performed using an unpaired two-tailed t test: ****$p < 0.0001$.

(K) Strong enrichment of Ca^{2+}-complexed pectins (labeled by the 2F4 antibody) around ITs (arrowheads) and transcellular passage sites (arrow). DNA was counterstained with DAPI (blue). TEM, transmission electron microscopy; SEM, scanning electron microscopy image.

Scale bars, 50 μm in (A)–(D); 2,5 μm in (E)–(F″); 10 μm in (G), (H), (I), and (K).

See also Figure S1.

Identification of symbiotically induced pectin methlyesterases

As pectin de-methylesterification is enzymatically mediated by pectin methyl esterases (PMEs),[12,15] we searched within the Medicago PME family, consisting of more than one hundred members, for symbiotic PMEs (SyPMEs) induced during RNS. We identified more than 30 SyPMEs that exhibit higher expression levels in the nodule meristem (fraction I; FI) and in cells of the infection zone (Figures S2A and S2B). Among those SyPMEs, one gene (Phyotozome: *Medtr4g087980* or *MtrunA17_Chr4g0069841*) was found to be additionally induced upon Nod factor (NF) application and *S. meliloti* inoculation in roots in several independent transcriptomic datasets (Figures S2C and S2D). Thus, we named the gene *SyPME1* and investigated it as a representative SyPME during rhizobial infection. We first verified the transcriptome data by *in situ* hybridizations and a histochemical assay expressing a *GUS* reporter under the control of a 2 kb fragment upstream of the start codon as a putative promoter region. When hybridizing nodule sections with an antisense *in situ* probe, *SyPME1* transcripts were found in cortical cells of nodule primordia (Figure S2F) and in zone zIId of mature nodules (Figure S2G). No signals were observed when using the sense probes as a control in the same tissues (Figures S2H and S1I). Accordingly, *SyPME1* promoter activity, as delineated by β-glucuronidase (GUS)-staining, was found within the entire cortex of nodule primordia (Figure S2J), whereas a confined expression domain limited to the infection zone II was observed in young and mature nodules (Figure S2K).

To assess the localization patterns of the SyPME1 protein, we initially generated a SyPME1-GFP translational fusion, which was driven by the native 2 kb *SyPME1* promoter fragment. Unfortunately, we were never able to obtain reliable fluorescent signals when using this construct in Medicago roots and nodules. However, when replacing the native promoter by a constitutively active Lotus *Ubiquitin 10* promoter (overexpression [OE]); SyPME1-OE), clear and confined fluorescence was observed in RHs around the ICs (Figure S3A) and along growing primary ITs (Figure S3B). In line with this, nodular ITs were also decorated by the SyPME1 protein (Figures 2A–2F), whereas the strongest accumulations were observed at

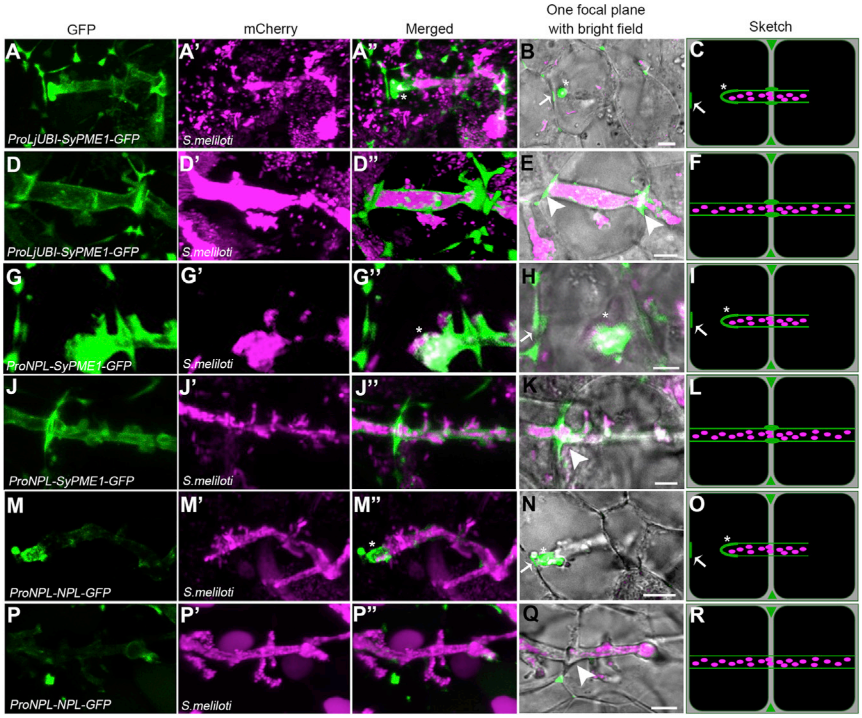

Figure 2. Spatially confined localization of the cell wall modifying enzymes SyPME1 and NPL

(A–R) SyPME1 driven by the *ProLjUBI* promoter (A)–(F) or the *ProNPL* promoter (G)–(L) in nodules 14 days post-inoculation with *S. meliloti* mCherry (magenta). SyPME1-GFP (green) accumulates strongly at the tip (asterisk) and moderately at the shanks of nodular ITs (A–C and G–I, n = 5) and remains at transcellular passage sites beyond IT progression (arrowheads; D–F and J–L, n = 20 for D–F and n = 15 for J–L). By contrast, NPL-GFP (green) predominantly accumulates at the tip of nodular ITs (asterisk; M–O, n = 5) but is absent from passage sites after IT progression (arrowhead, P–R, n = 15). Both proteins are also enriched at the passage site prior to IT arrival (arrow; B, H, and N; for close-up see Figures S3D–S3D', S3H– S3H', and S3L– S3L″). Scale bars, 5 μm. Images (A)–(A″), (D)–(D″), (G)–(G″), (J)–(J″), (M)–(M″), and (P)–(P″) were processed as 3D projections using the Imaris software package. The data were collected based on at least 3 independent rounds of hairy root transformation.
See also Figures S2 and S3.

transcellular passage sites (Figures 2D–2F). Here, SyPME1 localization was strictly delineated to the peripheral CW at cellular conjunctions with crossing ITs (Figures 2D and 2E), which are sites that are rich in Ca^{2+}-complexed un-esterified pectins (Figure 1K). Furthermore, we noticed that SyPME1 also accumulated at both the tip region of growing ITs and a spatially confined site at the cell periphery that marks the site of the subsequent transcellular IT passage (Figures 2A–2C, S3C, S3D, and S3D′). The same observation was also made using the symbiosis-inducible *NPL* promoter (*ProNPL*), which exhibits a similar expression profile compared with SyPME1 (Figure S2C and S2D), to drive the expression of *SyPME1* (Figures 2G–2L and S3E–S3H′).

To ensure that the *SyPME1* gene encodes an active PME, we determined its pectin methylesterase activity making use of the fact that the binding of ruthenium red to pectins increases with the removal of methyl esters.[16] For this, we obtained total protein extracts from inoculated Medicago roots transformed with the empty vector (EV) or a SyPME1-GFP (SyPME1-OE) construct. Incubation of these samples with ruthenium red revealed that global PME activity levels were significantly higher in the SyPME1-OE roots compared with control roots (Figure S2E), thus confirming that SyPME1 is an active PME.

NPL is required throughout the infection process

As un-esterified pectins serve as substrates for pectate lyases (PLs) or polygalacturonases (PGs), we investigated the role of Medicago NPL (Phytozome: Medtr3g086320 or MtrunA17_Chr3g0123331)[10,14] in more detail. Compared with other members of the PL family being present in nodule transcriptomic data,[17] *NPL* expression was found to be highest and mainly restricted to the nodule meristem (FI) and the zone zIId (Figure S2D). This spatially controlled expression in nodules was also confirmed when generating a transcriptional GUS reporter using 2 kb (2,038 bp) upstream of the *NPL* start codon (Figure S2L). On the protein level and in line with patterns observed for SyPME1, an NPL-GFP fusion protein driven by the endogenous *NPL* promoter also localized in ICs, primary ITs in RHs, and the above-mentioned spatially confined sites that will be penetrated by ITs (Figures S3I and S3K). Interestingly, older parts of these ITs showed reduced NPL accumulations (Figure S3J), whereas SyPME1 protein levels remained high in these regions (Figures S3B and S3F). Although NPL also localized to the tip of nodular ITs and local CW regions near nodular ITs (Figures 2M–2O and S3K–S3L′), the protein was absent from transcellular passage sites itself (Figures 2P–2R).

To analyze a possible cross-talk between SyPMEs and NPL in more detail, we genetically assessed their impact on infection using loss- and gain-of-function approaches. In line with data reported for the *Lotus japonicus* (Lotus) *npl* mutant,[14] significantly fewer nodules formed on the Medicago *npl* mutant compared with R108 WT plants (Figure 3A). Furthermore, most infection events were aborted at the IC stage (Figures 3B and 3C), an observation that is consistent with previously published data.[10] We then tested whether the loss of NPL had a direct consequence on pectin homeostasis in these mutants. Quantitative immuno-labeling of un-esterified and calcium-complexed pectins on IT CWs inside nodules and from the periphery of infected nodule cells revealed that the abundance of un-esterified (LM19, Figure 3D) and calcium-complexed pectins (2F4, Figure 3E) was significantly higher on nodular IT CWs of the *npl* mutant compared with the wild type (WT), whereas levels of esterified pectins on nodular CWs labeled by LM20 were unaltered in both genotypes, respectively (Figure 3F).

To be able to differentiate between the genetic requirement of NPL in the epidermis and the root cortex, we decided to additionally conduct an RNA interference (RNAi) approach and expressed

CellPress
OPEN ACCESS

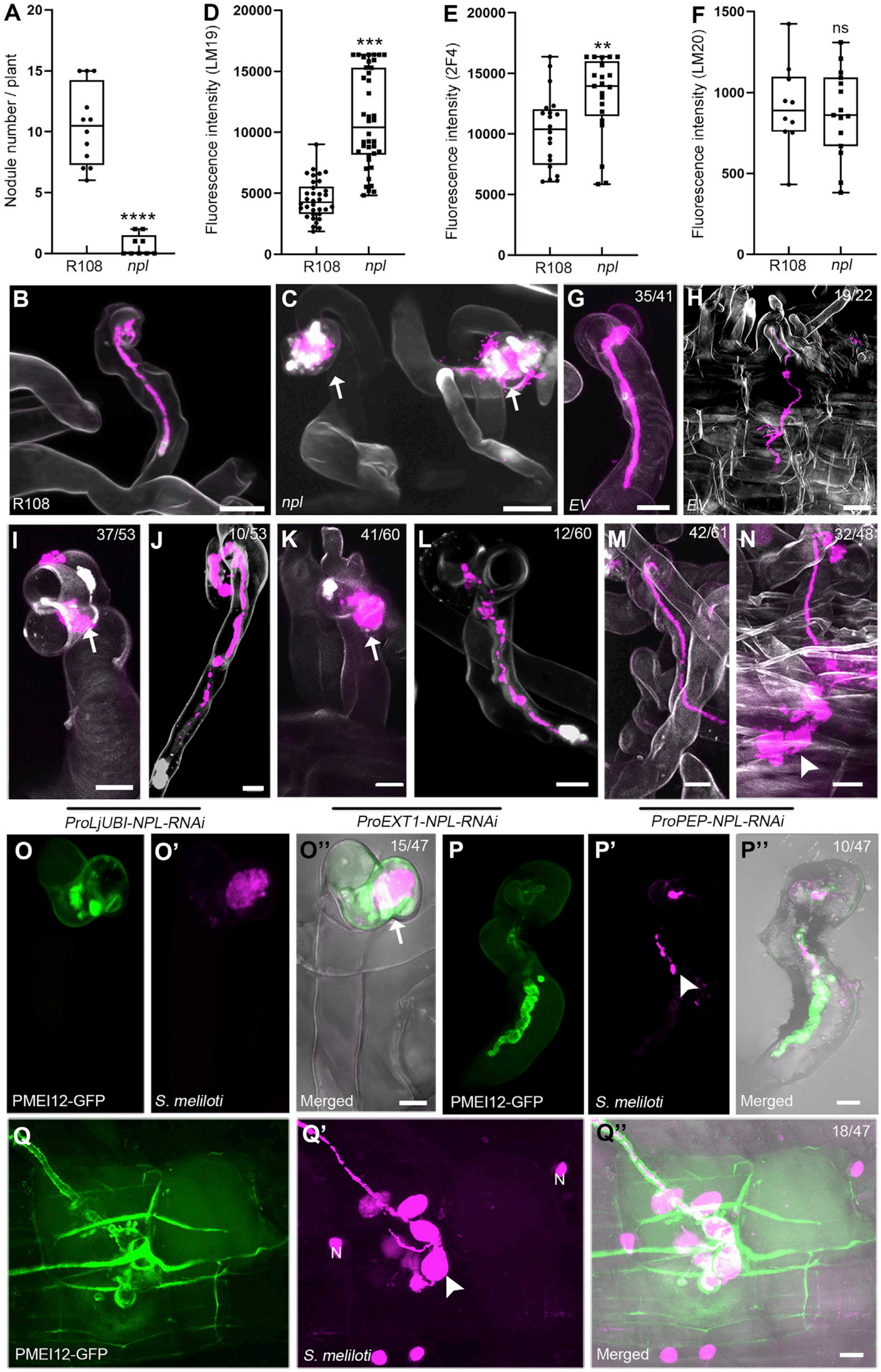

(legend on next page)

the silencing construct under the control of different tissue-specific promoters in transgenic roots. Although expression of an EV control resulted in the formation of WT-like ITs in RHs (Figure 3G) and their progression through the cortex (Figure 3H), ubiquitous expression of an NPL-RNAi construct resulted in frequent (37/53) entrapment of rhizobia within the IC (Figure 3I), whereas some ITs (10/ 53) managed to elongate but mostly aborted within the RHs (Figure 3J). To enable epidermal-specific expression, we cloned the *Solanum lycopersicum* (tomato) expansin 1 (*ProEXT1*) promoter that has previously been shown to mediate epidermis-specific expression in Medicago[18] and tested it by generating a GUS reporter. Roots carrying a *ProEXT1-GUS* reporter construct showed GUS activity in the root epidermis as well as in the endodermis (Figures S4A–S4A‴). Driving the NPL-RNAi construct under the control of *ProEXT1* led to an abortion of about 70% of all scored infections at the IC stage (41/60; Figure 3K), whereas in about 20% of the inspected roots, IT elongation was arrested within the RH (12/60; Figure 3L). For cortical expression, we assembled a GUS reporter driven by the *Arabidopsis thaliana* endopeptidase *PEP*-promoter (*ProPEP*) that has been previously used in Medicago.[19,20] We confirmed cortical activation of this promoter with the GUS signal being excluded from the epidermis and occasionally weaker in the outer compared with inner cortical cell layers (Figures S4B–S4B‴). When silencing *NPL* expression using a *ProPEP-NPL-RNAi* construct, we observed that most ITs successfully prolonged through the RHs (42/61; Figure 3M), whereas a large proportion of them aborted in the root cortex (32/48; Figure 3N).

We then wondered whether the loss of NPL results in ultrastructural changes of the IT CW. For this, we used our CLEM approach and labeled nodule sections obtained from WT and *ProPEP-NPL-RNAi* nodules for un-esterified pectins using the LM19 antibody (Figures S4C, S4C′, S4D, and S4D′). As observed for the *npl* mutant (Figure 3D), LM19 (against un-esterified pectin) signals obtained for the *ProPEP-NPL-RNAi* nodules were stronger than for the WT (Figure S4E). Scoring of CW thickness on these ITs revealed a significantly thicker CW in NPL-silenced nodules (1.301 ± 0.318 μm) compared with the WT (0.211 ± 0.016 μm) (Figures S4C″ and S4D″).

Taken together, these data confirmed that NPL not only serves functions during primary infection of RHs but also during transcellular passage within the root.

PME activity is required for IT progression

As mentioned above, global expression profiles of *NPL* and *SyPME1* are highly similar and both genes are significantly co-expressed during rhizobia infection (correlation coefficient = 0.9883, Figure S2C). To genetically test whether SyPME1 and/or other members of this protein family are required for successful infections, we first searched the Tnt1 transposon insertion collection and identified a single *SyPME1* allele carrying an insertion in the first exon (NF2281_high_35; Figure S4F). Homozygous individuals, however, did not show any symbiotic phenotypes (Figures S4G–S4K), which might be due to the large size and functional redundancy within this gene family (Figure S2A). In order to target functionally redundant SyPMEs with spatiotemporal precision, we expressed the *Arabidopsis thaliana PME INHIBITOR 12* (*PMEI12*) under the control of the *NPL* promoter and inoculated these transgenic roots with *S. meliloti*. This protein has been previously demonstrated to efficiently inhibit PME activities.[21,22] We also confirmed this under our conditions in the same ruthenium red-based gel diffusion assay that we used to show the esterase activity of SyPME1 (Figure S2E). Indeed, the expression of PMEI12 reduced overall PME activities moderately but significantly compared with the EV control (Figure S2E). When phenotyping PMEI12-expressing roots, we observed that the most infections were prematurely blocked (43/47) either at the IC stage (15/47; Figures 3O–3O″) or during IT progression in RHs (10/47; Figures 3P–3P″) and the root cortex (18/47; Figures 3Q–3Q″), thus phenocopying the *npl* mutant. This supports the proposed dependency of NPL-mediated pectin degradation on preceding PME activity.

Deciphering the steps of IT passage by CLEM

In a last set of experiments and to unambiguously unravel CW changes during transcellular IT passage, we used our CLEM approach to monitor pectin alterations within the cell-cell interface at different stages of transcellular IT progression. For this,

Figure 3. NPL activity is required throughout IT progression in different cell layers

(A–C) The *npl* mutant developed significantly fewer nodules compared with the R108 (WT) control when grown for 7 days in the presence of *S. meliloti* in open pots (A; R108: n = 12, *npl*: n = 9) with the majority of infections already being blocked at the root hair stage (B) and (C). Data are mean ± SE. Statistics were performed using an unpaired two-tailed t test: ****p < 0.0001.

(D–F) Quantitative immuno-labeling of un-esterified pectins (D, n = 34 for R108 and n = 40 for *npl*), calcium cross-linked pectins (E, n = 20 for both R108 and *npl*), and esterified pectins (F, n = 10 for R108 and n = 15 for *npl*). The fluorescence intensity was measured from IT cell walls for LM19 and 2F4; for LM20 the fluorescence intensity was measured from nodule cell walls. For each condition, sections from 5 nodules were used for analysis and measure multi-regions on each section. Data are mean ± SE. Statistics were performed using an unpaired two-tailed t test: ***p < 0.001, **p < 0.01, ns, not significant.

(G and H) WT-like IT growth in composite roots (G) and cortical cells (H) of control roots transformed with an empty vector (EV).

(I and J) Composite roots expressing an RNA interference (RNAi) construct against NPL (*ProLjUBI-NPL-RNAi*) showed aborted infections around the infection chamber (arrows; I), whereas some IT managed to elongate but aborted within the root hairs (J).

(K and L) The same effect was observed upon epidermis-specific expression of the RNAi construct (*ProEXT1-NPL-RNAi*).

(M and N) Silencing *NPL* expression in root cortical cells (*ProPEP-NPL-RNAi*) did not impair IT growth in root hairs (M) but transcellular progression in the root cortex (N).

(O–Q″) Expression of PMEI12-GFP under the control of the *NPL* promoter resulted in IT abortions around the infection chamber (O–O″, the green signal indicates PMEI-GFP accumulations around the infection chamber [IC]; the arrow indicates enlarged IC), in root hairs (P–P″, the green signal indicates the PMEI-GFP accumulation around the infection thread [IT] and the arrow indicates an aborted IT), and in the root cortex (Q–Q″, green signal is the PMEI-GFP located to the IT cell wall and the cell wall from host cortical cells containing an IT and the neighboring cells; the arrow indicates an aborted IT in a root cortical cell). The numbers indicate the observed events. N, nucleus; scale bars, 10 μm.

All images (B)–(Q″) are with maximal projection. All the experiments were performed with 3 biological replicates and showed similar results.

See also Figure S4.

CellPress
OPEN ACCESS

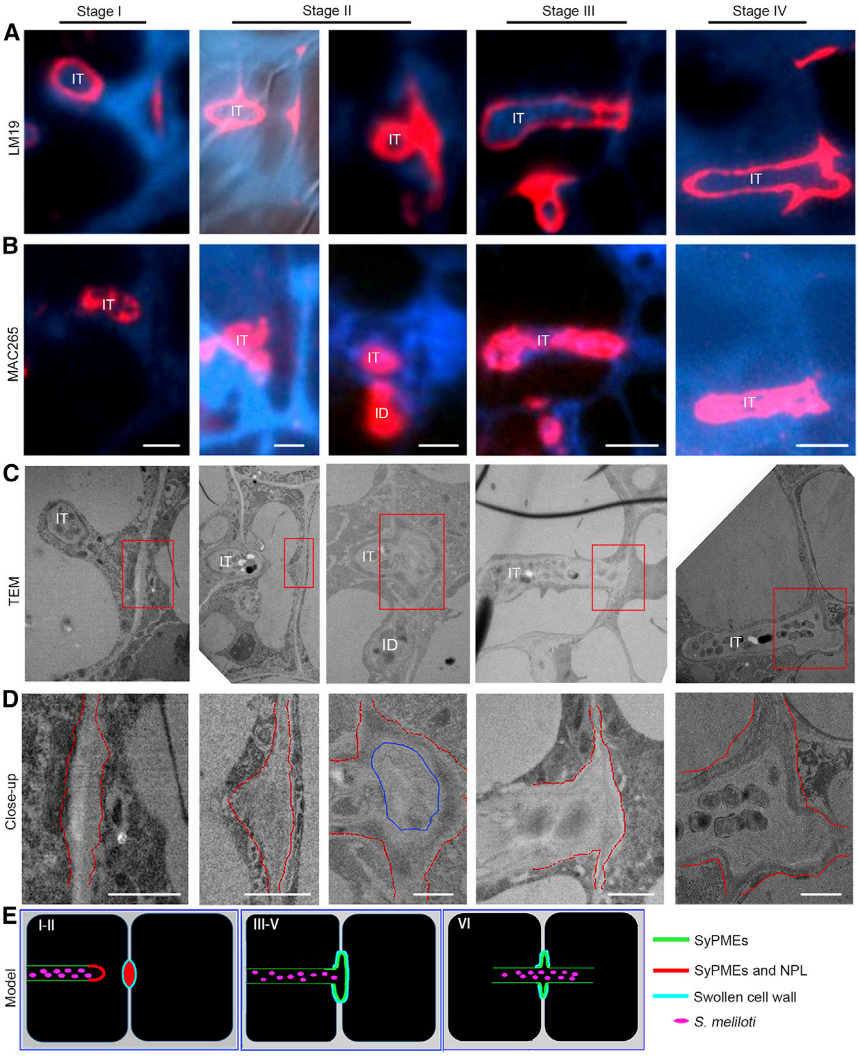

Figure 4. CLEM analysis of distinct steps during transcellular IT passage

(A and B) CLEM analysis for cell wall modifications at the IT (infection thread) penetration sites for un-esterified pectins (labeled by LM19 (red), (A) and IT matrix components (labeled by MAC265 (red); (B) with consecutive sections.

(C and D) (C) Corresponding TEM micrographs and their close-ups (D) as indicated by the red boxes in (C). Red dashed lines (in D) indicate the border of the cell wall; the blue encircled region indicates the cell wall swelled in the central region.

(E) Proposed graphical model for different stages of IT transcellular passage.

Scale bars, 4 μm in (A)–(C) and 1 μm in (D). ID, infection droplet. All the experiments were performed with nodule samples at least harvested from 2 independent biological replicates.

See also Figure S4.

we labeled un-esterified pectins (LM19, Figure 4A) and the IT matrix (MAC265, Figure 4B) and searched ultrathin sections (Figures 4C and 4D) for ITs approaching the basal cell membrane/CW and the moment of IT passage. Un-esterified pectins accumulated around the future transcellular passage site, as defined by the cytoplasmatic column formed ahead of the IT, within the cell-cell interface (Figure 4, stage I). This passage site further increased in size, expanded laterally, and subsequently swelled in the central region (Figure 4, stage II). This was followed by rhizobia entering a closed apoplastic compartment prior to entry into the neighboring cell that had already formed a pre-IT (Figure 4, stage III). At the end, rhizobia entered the neighboring cell through the apoplastic space without any membrane confinement at this site (Figure 4, stage IV).

Given the estimated diameter of the passage site in the range between 1 and 3 μm, the discontinuation of the IT membrane upon fusion of the IT with the basal membrane, and the *de novo* invagination at the neighboring cell, we conclude that transcellular passage is a successive series of events (Figure 4E). This includes (1) targeted secretion of SyPMEs and initially of NPL, (2) local pectin de-methylesterification and partial pectin degradation at the tip of the IT and at the local host CW prepared for penetration, (3) maintenance of SyPMEs but the reduction of NPL protein levels at the passage site, (4) confined CW swelling at the passage site, (5) the spatial release of rhizobia into a sealed apoplastic compartment, and (6) uptake into the neighboring cell.

DISCUSSION

The intracellular colonization of differentiating nodule primordium cells is a conserved process in model legumes such as Lotus and Medicago as well as in most agriculturally relevant legume species including soybean, pea, and beans. This phenomenon requires the transcellular passage of the IT throughout several cell layers. Although it has been assumed decades ago that ITs pass the CW via apertures around the middle lamella,[23] it has been proposed that this does not involve CW pits (plasmodesmata).[24] This was further supported by the fact that the IT membrane fuses with the basal plasma membrane of the host cell upon transcellular passage.[25] In indeterminate nodulators such as Medicago, transcellular progression of ITs does not only occur in the outer root cortex but also constantly in the distal part of the so-called nodular "infection zone."[2] Here, we often observed a local thickening of the CW (Figure 4C) at the future passage sites, although we never detected the formation of true CW apertures at these sites of IT penetration. Additionally, both SyPME1 and NPL accumulated at the prospective passage sites prior to IT arrival at the plasma membrane (Figures 2B, 2H, 2N, S3C–S3D′, S3G–S3H′, and S3K–S3L″), which indicates that a spatially confined and partial pectin degradation is involved in preparing these sites for penetration by generating a weakened CW region. Subsequently, CW swelling at the penetration site (Figure 4) might be induced by PME-mediated de-methylesterification of pectins, which can

lead to CW expansion and hydration.[26] It should be noted that this process, alike the formation of egg-box pectin, also requires Ca^{2+}-complexation.[27,28] Consequently, the presence of complexed un-esterified pectins, as labeled by the LM19 and 2F4 antibodies (Figure 1) around the transcellular IT passage sites, would result in local stiffening or swelling of the CW in the absence of NPL at these sites (Figures 2P–2Q). The resulting swelling of the cell-cell interface may be further supported by a low cellular turgor pressure in recently divided nodule cells that are the ones that can be successfully entered by the IT[29] and by the action of expansin proteins. These apoplastic proteins support CW loosening without having any known enzymatic activity.[30] Indeed, apoplastic accumulation of expansins around ITs has been reported in pea[31] and positively regulates nodulation in soybean.[32] Similar CW swellings were also observed during colonization of roots by symbiotic arbuscular mycorrhiza fungi[33] and during stomata pore formation.[34] Although the initial processes might be comparable with those described here for rhizobial infections, the final stomatal pore is formed by the continuous accumulation of de-esterified pectins and PG PGX3/1 activity.[35] During transcellular IT passage, however, NPL only transiently accumulates prior to and upon the IT reaching the basal membrane, whereas SyPMEs accumulate throughout the process and remains present at this site (Figure 2). The subsequent stiffening as indicated by an accumulation of egg-box pectin (Figure 1K) may ensure the local confinement of the rhizobia in the apoplastic space by sealing this site from the surrounding.

The role of NPL and SyPMEs in controlling such local CW texture and thickness is genetically supported as cortical silencing of NPL resulted in a significantly thickened IT wall (Figures S4D–S4D″), which, most likely triggers premature IT abortion as observed in roots expressing this silencing construct (Figure 3N). Furthermore, it is interesting to note that accumulations of NPL may be under tight spatiotemporal control as NPL accumulated at the IT tip and the basal CW prior to IT passage (Figures 2M–2O) but did not remain at the passage site itself (Figures 2P–2R). The genetic dissection of PME activity, was, however more difficult, probably due to functional redundancy within the large PME family. This hypothesis is supported by the findings that expression of the PME inhibitor 12 from a symbiosis-induced promoter resulted in more IT abortions (Figures 3O–3Q″) and that extracts from roots expressing PMEI12 lowered but not fully abolished PME activity in our in vitro assay (Figure S2E). Similar to NPL, SyPME1 protein accumulations were tightly confined as focal accumulations at the IT tip and the pre-penetration site despite the over-expression of the protein. In addition, and differently from NPL, SyPME1 was consistently maintained at transcellular passage sites (Figures 2D, 2E, 2J, and 2K). This specificity could be maintained by a well-orchestrated targeted secretion and consequently an intact cytoskeleton. The latter may be controlled by the SCAR protein API since the *api* mutant exhibits alterations in CW properties[36] and in which the majority of ITs is blocked in root cortex cells prior to nodule primordium invasion.[37]

Taken together, we conclude that SyPMEs and NPL are initially targeted to the same sites but that SyPME function precedes NPL-mediated pectin degradation at the tip region of growing ITs and initially at the local CW site preparing for IT passage. NPL activity in turn prevents stiffening of the IT tip and enables local CW loosening prior to IT arrival at the basal cell membrane. Transcellular passage and bacterial confinement are then enabled by pectin complexation that seals this distinct site. This enzymatic interplay may thus allow the growth of the IT through several cell layers and rhizobial isolation in the apoplastic space.

STAR★METHODS

Detailed methods are provided in the online version of this paper and include the following:

- KEY RESOURCES TABLE
- RESOURCE AVAILABILITY
  - Lead Contact
  - Materials Availability
  - Data and code availability
- EXPERIMENTAL MODEL AND SUBJECT DETAILS
- METHOD DETAILS
  - Plant growth and hairy root transformation
  - Phenotyping
  - Visualization of IT phenotypes
  - *In situ* hybridization
  - Cloning and DNA constructs
  - GUS staining
  - Immunofluorescence labelling
  - Fluorescence microscopy
  - Sample preparation for TEM analysis
  - Sample preparation for CLEM
  - Immuno-Gold staining
  - Determination of total PME activities
- QUANTIFICATION AND STATISTICAL ANALYSIS

SUPPLEMENTAL INFORMATION

Supplemental information can be found online at https://doi.org/10.1016/j.cub.2022.12.051.

ACKNOWLEDGMENTS

We would like to thank Eija Schulze and Carmen Schubert for their experimental support and technical help and the entire Ott lab team and Elke Barbez for the continuous discussions on the project. Seeds from *npl* and *cbs* mutants were provided by Chengwu Liu (University of Science and Technology of China) and Michael Udvardi (The University of Queensland, Australia). We also thank the staff of the Life Imaging Center (LIC) in the Hilde Mangold House (HMH) of the Albert-Ludwigs-University of Freiburg for help with their confocal microscopy resources and the excellent support in image recording. The microscopes are operated by the Microscopy and Image Analysis Platform (MIAP) and the Life Imaging Center (LIC), Freiburg. Norbert Roos and Jens Wohlmann (Electron Microscopy Facility, Department of Biosciences, University of Oslo, Norway) are acknowledged for helping with the SEM sample preparation and imaging. The *Medicago truncatula* plants utilized in this research project, which are jointly owned by the Centre National De La Recherche Scientifique, were obtained from Noble Research Institute, LLC and were created through research funded, in part, by a grant from the National Science Foundation, NSF-0703285. This study was funded by: the Engineering Nitrogen Symbiosis for Africa (ENSA) project currently supported through a grant to the University of Cambridge by the Bill & Melinda Gates Foundation (OPP1172165) and the UK Government's Department for International Development (DFID) (T.O.), the Deutsche Forschungsgemeinschaft (DFG, German Research Foundation) grant 431626755 (X.L. and T.O.), the National Natural

Science Foundation of China (NSFC, National Natural Science Foundation of China) 31961133029 (X.L. and T.O.), the DFG under Germany's Excellence Strategy grant CIBSS – EXC-2189 – project ID 39093984 (T.O.), the China Scholarship Council (CSC) grants 201708080016 (C.S.) and 202006300033 (G.Z.), and the DFG equipment grants 414136422 (CLSM; T.O.) and 426849454 (TEM; T.O.).

AUTHOR CONTRIBUTIONS

Conceptualization, C.S., X.L., and T.O.; investigation, C.S., G.Z., M.R.-F., J.W., R.H., W.Y., P.L., and L.U.; writing – original draft, C.S. and T.O.; writing – review & editing, C.S., G.Z., M.R.-F., J.W., W.Y., P.L., L.U., X.L., and T.O.; supervision, C.S., X.L., and T.O.; project administration, X.L. and T.O.; funding acquisition, C.S., G.Z., X.L., and T.O.

DECLARATION OF INTERESTS

The authors declare that a patent application has been submitted (U.S. Provisional Application Serial No. 63/367,703).

Received: June 3, 2022
Revised: November 18, 2022
Accepted: December 20, 2022
Published: January 18, 2023

REFERENCES

1. Fournier, J., Teillet, A., Chabaud, M., Ivanov, S., Genre, A., Limpens, E., de Carvalho-Niebel, F., and Barker, D.G. (2015). Remodeling of the infection chamber before infection thread formation reveals a two-step mechanism for rhizobial entry into the host legume root hair. Plant Physiol. *167*, 1233–1242. https://doi.org/10.1104/pp.114.253302.
2. Gage, D.J. (2004). Infection and invasion of roots by symbiotic, nitrogen-fixing rhizobia during nodulation of temperate legumes. Microbiol. Mol. Biol. Rev. *68*, 280–300. https://doi.org/10.1128/MMBR.68.2.280-300.2004.
3. Fournier, J., Timmers, A.C., Sieberer, B.J., Jauneau, A., Chabaud, M., and Barker, D.G. (2008). Mechanism of infection thread elongation in root hairs of Medicago truncatula and dynamic interplay with associated rhizobial colonization. Plant Physiol. *148*, 1985–1995. https://doi.org/10.1104/pp.108.125674.
4. Gaudioso-Pedraza, R., Beck, M., Frances, L., Kirk, P., Ripodas, C., Niebel, A., Oldroyd, G.E.D., Benitez-Alfonso, Y., and de Carvalho-Niebel, F. (2018). Callose-regulated symplastic communication coordinates symbiotic root nodule development. Curr. Biol. *28*. 3562.e6–3577.e6. https://doi.org/10.1016/j.cub.2018.09.031.
5. Voragen, A.G.J., Coenen, G.J., Verhoef, R.P., and Schols, H.A. (2009). Pectin, a versatile polysaccharide present in plant cell walls. Struct. Chem. *20*, 263–275. https://doi.org/10.1007/s11224-009-9442-z.
6. Vandenbosch, K.A., Bradley, D.J., Knox, J.P., Perotto, S., Butcher, G.W., and Brewin, N.J. (1989). Common components of the infection thread matrix and the intercellular space identified by immunocytochemical analysis of pea nodules and uninfected roots. EMBO J. *8*, 335–341. https://doi.org/10.1002/j.1460-2075.1989.tb03382.x.
7. Tsyganova, A.V., Seliverstova, E.V., Brewin, N.J., and Tsyganov, V.E. (2019). Comparative analysis of remodelling of the plant-microbe interface in Pisum sativum and Medicago truncatula symbiotic nodules. Protoplasma *256*, 983–996. https://doi.org/10.1007/s00709-019-01355-5.
8. Sujkowska-Rybkowska, M., and Borucki, W. (2014). Accumulation and localization of extensin protein in apoplast of pea root nodule under aluminum stress. Micron *67*, 10–19. https://doi.org/10.1016/j.micron.2014.06.006.
9. Gavrin, A., Chiasson, D., Ovchinnikova, E., Kaiser, B.N., Bisseling, T., and Fedorova, E.E. (2016). VAMP721a and VAMP721d are important for pectin dynamics and release of bacteria in soybean nodules. New Phytol. *210*, 1011–1021. https://doi.org/10.1111/nph.13837.
10. Liu, C.W., Breakspear, A., Stacey, N., Findlay, K., Nakashima, J., Ramakrishnan, K., Liu, M., Xie, F., Endre, G., de Carvalho-Niebel, F., et al. (2019). A protein complex required for polar growth of rhizobial infection threads. Nat. Commun. *10*, 2848. https://doi.org/10.1038/s41467-019-10029-y.
11. Nguema-Ona, E., Vicré-Gibouin, M., Cannesan, M.A., and Driouich, A. (2013). Arabinogalactan proteins in root-microbe interactions. Trends Plant Sci. *18*, 440–449. https://doi.org/10.1016/j.tplants.2013.03.006.
12. Pelloux, J., Rustérucci, C., and Mellerowicz, E.J. (2007). New insights into pectin methylesterase structure and function. Trends Plant Sci. *12*, 267–277. https://doi.org/10.1016/j.tplants.2007.04.001.
13. Wormit, A., and Usadel, B. (2018). The multifaceted role of pectin methylesterase inhibitors (PMEIs). Int. J. Mol. Sci. *19*, 2878. https://doi.org/10.3390/ijms19102878.
14. Xie, F., Murray, J.D., Kim, J., Heckmann, A.B., Edwards, A., Oldroyd, G.E., and Downie, J.A. (2012). Legume pectate lyase required for root infection by rhizobia. Proc. Natl. Acad. Sci. USA *109*, 633–638. https://doi.org/10.1073/pnas.1113992109.
15. Jolie, R.P., Duvetter, T., Van Loey, A.M., and Hendrickx, M.E. (2010). Pectin methylesterase and its proteinaceous inhibitor: a review. Carbohydr. Res. *345*, 2583–2595. https://doi.org/10.1016/j.carres.2010.10.002.
16. Downie, B., Dirk, L.M., Hadfield, K.A., Wilkins, T.A., Bennett, A.B., and Bradford, K.J. (1998). A gel diffusion assay for quantification of pectin methylesterase activity. Anal. Biochem. *264*, 149–157. https://doi.org/10.1006/abio.1998.2847.
17. Roux, B., Rodde, N., Jardinaud, M.F., Timmers, T., Sauviac, L., Cottret, L., Carrère, S., Sallet, E., Courcelle, E., Moreau, S., et al. (2014). An integrated analysis of plant and bacterial gene expression in symbiotic root nodules using laser-capture microdissection coupled to RNA sequencing. Plant J. *77*, 817–837. https://doi.org/10.1111/tpj.12442.
18. Rival, P., de Billy, F., Bono, J.J., Gough, C., Rosenberg, C., and Bensmihen, S. (2012). Epidermal and cortical roles of NFP and DMI3 in coordinating early steps of nodulation in Medicago truncatula. Development *139*, 3383–3391. https://doi.org/10.1242/dev.081620.
19. Ron, M., Kajala, K., Pauluzzi, G., Wang, D.X., Reynoso, M.A., Zumstein, K., Garcha, J., Winte, S., Masson, H., Inagaki, S., et al. (2014). Hairy root transformation using Agrobacterium rhizogenes as a tool for exploring cell type-specific gene expression and function using tomato as a model. Plant Physiol. *166*, 455–469. https://doi.org/10.1104/pp.114.239392.
20. Sevin-Pujol, A., Sicard, M., Rosenberg, C., Auriac, M.C., Lepage, A., Niebel, A., Gough, C., and Bensmihen, S. (2017). Development of a GAL4-VP16/UAS trans activation system for tissue specific expression in Medicago truncatula. PLoS One *12*, e0188923. https://doi.org/10.1371/journal.pone.0188923.
21. An, S.H., Sohn, K.H., Choi, H.W., Hwang, I.S., Lee, S.C., and Hwang, B.K. (2008). Pepper pectin methylesterase inhibitor protein CaPMEI1 is required for antifungal activity, basal disease resistance and abiotic stress tolerance. Planta *228*, 61–78. https://doi.org/10.1007/s00425-008-0719-z.
22. Lionetti, V., Fabri, E., De Caroli, M., Hansen, A.R., Willats, W.G.T., Piro, G., and Bellincampi, D. (2017). Three pectin methylesterase inhibitors protect cell wall integrity for *Arabidopsis* immunity to Botrytis. Plant Physiol. *173*, 1844–1863. https://doi.org/10.1104/pp.16.01185.
23. McCoy, E., and Russell, E.J. (1932). Infection by *bact*. *radicicola* in relation to the microchemistry of the host's cell walls. Proc. R. Soc. Lond. B. *110*, 514–533. https://doi.org/10.1098/rspb.1932.0040.
24. Goodchild, D.J., and Bergersen, F.J. (1966). Electron microscopy of the infection and subsequent development of soybean nodule cells. J. Bacteriol. *92*, 204–213. https://doi.org/10.1128/jb.92.1.204-213.1966.
25. Szczyglowski, K., Shaw, R.S., Wopereis, J., Copeland, S., Hamburger, D., Kasiborski, B., Dazzo, F.B., and De Bruijn, F.J. (1998). Nodule organogenesis and symbiotic mutants of the model legume Lotus japonicus. Mol. Plant Microbe Interact. *11*, 684–697.

26. Peaucelle, A., Wightman, R., and Höfte, H. (2015). The control of growth symmetry breaking in the *Arabidopsis* hypocotyl. Curr. Biol. *25*, 1746–1752. https://doi.org/10.1016/j.cub.2015.05.022.

27. White, P.B., Wang, T., Park, Y.B., Cosgrove, D.J., and Hong, M. (2014). Water-polysaccharide interactions in the primary cell wall of *Arabidopsis thaliana* from polarization transfer solid-state NMR. J. Am. Chem. Soc. *136*, 10399–10409. https://doi.org/10.1021/ja504108h.

28. Wang, T., Park, Y.B., Cosgrove, D.J., and Hong, M. (2015). Cellulose-pectin spatial contacts are inherent to never-dried Arabidopsis primary cell walls: evidence from solid-state nuclear magnetic resonance. Plant Physiol. *168*, 871–884. https://doi.org/10.1104/pp.15.00665.

29. Monahan-Giovanelli, H., Pinedo, C.A., and Gage, D.J. (2006). Architecture of infection thread networks in developing root nodules induced by the symbiotic bacterium Sinorhizobium meliloti on Medicago truncatula. Plant Physiol. *140*, 661–670. https://doi.org/10.1104/pp.105.072876.

30. Cosgrove, D.J. (2016). Plant cell wall extensibility: connecting plant cell growth with cell wall structure, mechanics, and the action of wall-modifying enzymes. J. Exp. Bot. *67*, 463–476. https://doi.org/10.1093/jxb/erv511.

31. Sujkowska, M., Borucki, W., and Golinowski, W. (2007). Localization of expansin-like protein in apoplast of pea (Pisum sativum L.) root nodules during interaction with Rhizobium leguminosarum bv. Viciae 248. Acta Soc. Bot. Pol. *76*, 17–26.

32. Li, X.X., Zhao, J., Tan, Z.Y., Zeng, R.S., and Liao, H. (2015). GmEXPB2, a cell wall beta-expansin, affects soybean nodulation through modifying root architecture and promoting nodule formation and development. Plant Physiol. *169*, 2640–2653. https://doi.org/10.1104/pp.15.01029.

33. Rich, M.K., Schorderet, M., and Reinhardt, D. (2014). The role of the cell wall compartment in mutualistic symbioses of plants. Front. Plant Sci. *5*, 238. https://doi.org/10.3389/fpls.2014.00238.

34. Nadeau, J.A., and Sack, F.D. (2002). Control of stomatal distribution on the *Arabidopsis* leaf surface. Science *296*, 1697–1700. https://doi.org/10.1126/science.1069596.

35. Rui, Y., Xiao, C.W., Yi, H.J., Kandemir, B., Wang, J.Z., Puri, V.M., and Anderson, C.T. (2017). Polygalacturonase INVOLVED IN EXPANSION3 functions in seedling development, rosette growth, and stomatal dynamics in *Arabidopsis thaliana*. Plant Cell *29*, 2413–2432. https://doi.org/10.1105/tpc.17.00568.

36. Gavrin, A., Rey, T., Torode, T.A., Toulotte, J., Chatterjee, A., Kaplan, J.L., Evangelisti, E., Takagi, H., Charoensawan, V., Rengel, D., et al. (2020). Developmental modulation of root cell wall architecture confers resistance to an oomycete pathogen. Curr. Biol. *30*. 4165.e5–4176.e5. https://doi.org/10.1016/j.cub.2020.08.011.

37. Teillet, A., Garcia, J., de Billy, F., Gherardi, M., Huguet, T., Barker, D.G., de Carvalho-Niebel, F., and Journet, E.P. (2008). api, a novel Medicago truncatula symbiotic mutant impaired in nodule primordium invasion. Mol. Plant Microbe Interact. *21*, 535–546. https://doi.org/10.1094/MPMI-21-5-0535.

38. Schindelin, J., Arganda-Carreras, I., Frise, E., Kaynig, V., Longair, M., Pietzsch, T., Preibisch, S., Rueden, C., Saalfeld, S., Schmid, B., et al. (2012). Fiji: an open-source platform for biological-image analysis. Nat. Methods *9*, 676–682. https://doi.org/10.1038/nmeth.2019.

39. Boisson-Dernier, A., Chabaud, M., Garcia, F., Bécard, G., Rosenberg, C., and Barker, D.G. (2001). Agrobacterium rhizogenes-transformed roots of Medicago truncatula for the study of nitrogen-fixing and endomycorrhizal symbiotic associations. Mol. Plant Microbe Interact. *14*, 695–700. https://doi.org/10.1094/MPMI.2001.14.6.695.

40. Jackson, A.C. (1992). Detection of rabies virus mRNA in mouse brain by using in situ hybridization with digoxigenin-labelled RNA probes. Mol. Cell. Probes *6*, 131–136. https://doi.org/10.1016/0890-8508(92)90057-5.

41. Weber, E., Engler, C., Gruetzner, R., Werner, S., and Marillonnet, S. (2011). A modular cloning system for standardized assembly of multigene constructs. PLoS One *6*, e16765. https://doi.org/10.1371/journal.pone.0016765.

42. Su, C., Klein, M.L., Hernández-Reyes, C., Batzenschlager, M., Ditengou, F.A., Lace, B., Keller, J., Delaux, P.M., and Ott, T. (2020). The Medicago truncatula DREPP protein triggers microtubule fragmentation in membrane nanodomains during symbiotic infections. Plant Cell *32*, 1689–1702. https://doi.org/10.1105/tpc.19.00777.

43. Xue, J., Bosch, M., and Knox, J.P. (2013). Heterogeneity and glycan masking of cell wall microstructures in the stems of Miscanthus x giganteus, and its parents M. sinensis and M. sacchariflorus. PLoS One *8*, e82114. https://doi.org/10.1371/journal.pone.0082114.

CellPress
OPEN ACCESS

STAR★METHODS

KEY RESOURCES TABLE

REAGENT or RESOURCE	SOURCE	IDENTIFIER
**Antibodies**		
MAC265 Rat monoclonal: Infection thread matrix glycoprotein	CarboSource	http://glycomics.ccrc.uga.edu/wall2/antibodies/antibodyHome.html
LM5 Rat monoclonal: Pectic polysaccharide ((1-4)-β-D-galactan)	PlantProbes	https://plantcellwalls.leeds.ac.uk/science/antibodies/
LM6 Rat monoclonal: Pectic polysaccharide ((1-5)-α-L-arabinosyl)	PlantProbes	https://plantcellwalls.leeds.ac.uk/science/antibodies/
LM19 Rat monoclonal: Un-esterified homogalacturonan	PlantProbes	https://plantcellwalls.leeds.ac.uk/science/antibodies/ RRID:AB_2734788
LM20 Rat monoclonal: Methyl-esterified homogalacturonan	PlantProbes	https://plantcellwalls.leeds.ac.uk/science/antibodies/ RRID:AB_2734789
2F4 Mouse monoclonal: ’egg box’’ dimer conformation of homogalacturonan	PlantProbes	http://glycomics.ccrc.uga.edu/wall2/antibodies/antibodyHome.html
LM2 Rat monoclonal: Arabinogalactan-proteins (AGPs)	PlantProbes	https://plantcellwalls.leeds.ac.uk/science/antibodies/
LM14 Rat monoclonal: Arabinogalactan-proteins (AGPs)	PlantProbes	https://plantcellwalls.leeds.ac.uk/science/antibodies/
LM30 Rat monoclonal: Arabinogalactan-proteins (AGPs)	PlantProbes	https://plantcellwalls.leeds.ac.uk/science/antibodies/
LM25 Rat monoclonal: Xyloglucan	PlantProbes	https://plantcellwalls.leeds.ac.uk/science/antibodies/
AlexaFluor 546 goat anti-rat	Thermo Fisher	RRID:AB_2534125
AlexaFluor 546 goat anti-mouse	Thermo Fisher	RRID:AB_2534071
Protein A-gold 5 nm	PAG5; CMC, Utrecht, The Netherlands	N/A
Goat-anti Rat IgG 12 nm gold	Jackson Immuno Research	RRID:AB_2338272
**Bacterial and virus strains**		
Sinorhizobium meliloti 2011	Lab stocks	N/A
Agrobacterium rhizogenes strain ARqua1	Lab stocks	N/A
**Chemicals, peptides, and recombinant proteins**		
Calcofluor White	Fluka	Cat#18909-100ML-F
X-Gluc (5-bromo-4-chloro-3-indoxyl-b-D-GlcA, cyclohexylammonium salt	Carl Roth	Cat#Art.Nr.0018.3
Ruthenium red	Sigma-Aldrich	Cat#R2751
Toluidine blue	Sigma-Aldrich	Cat#89640
glutaraldehyde 25%	Carl Roth	Art.Nr.4157.3
Paraformaldehyde (PFA)	Sigma-Aldrich	Cat#P-6148
xyloglucanase (XYLOGLUCANASE (GH5) (Paenibacillus sp.)	Megazyme	Cat#no. E-XEGP
PECTATE LYASE (Apergillus sp.)	Megazyme	Cat#no. E-PCL YAN
**Critical commercial assays**		
Bsa I enzyme for Goldengate reactions	New England BIOLABS	Cat#R3733
Bpi I enzyme for Goldengate reactions	New England BIOLABS	Cat#R35395
Technovit 7100	MORPHISTO	Cat#12227.K0500
Technovit 8100	MORPHISTO	Cat#12226.K0500

(*Continued on next page*)

Continued

REAGENT or RESOURCE	SOURCE	IDENTIFIER
**Experimental models: Organisms/strains**		
Medicago truncatula cultivar Jemalong	Heritage Seeds Pty, Adelaide, AU	Jemalong
Medicago truncatula ecotype R108 Tnt1 insertion line (*sypme1*)	*Medicago truncatula* Mutant Database: https://medicago-mutant.dasnr.okstate.edu/mutant/index.php	NF2281
Medicago truncatula ecotype R108 Tnt1 insertion line (*npl*)	Rival et al.[18]	NF18556
**Oligonucleotides**		
Primers for genotyping, SYMPEM-F: CTCTACACAACTGA ATCTACAATCTTTTCC	This paper	N/A
Primers for genotyping, SYMPEM-R: CAGAATATTCTATCATA ACCCAATGCAACC	This paper	N/A
Primers for genotyping, TNT-R: TGTAGCACCGAGATA CGGTAATTAACAAGA	This paper	N/A
Primers for in situ, SyPME1-situ-F: TGTGTTTGGTC ACTCTCGCAC	This paper	N/A
Primers for in situ, SyPME1-situ-R: ACAATTGAACCTTCT AATCCTTCTATACAT	This paper	N/A
Sequence for NPL-RNAi design: TCGGTAGACGTGCAATT GGAGGTAAAGATGGAAAA TATTACATGGTCATTGACT CAAGTGATGACCCTGTGA ATCCTAAGCCAGGAACATT AAGACATGCTGTTATCCAACAA GAGCCTTTATGGATCATTTTCAA GCATGACATGGTGATCAAACT AAAGATGGATCTTCTCATGAAT TCTTTCAAAACAATTGATGGTA GAGGTGTAAATGTACACATTGCT GAAGGACCATGTATTAGAATACA AGAAAAGACCAACATCATAATT CATGGTATACACATTCA TCATTGTGTACGAG	This paper	N/A
**Recombinant DNA**		
Promoter activity:ProNPL::GUS//ProUBI::NLS-2xmCherry	This paper	N/A
Promoter activity: ProSyPME1::GUS//ProUBI::NLS-2xmCherry	This paper	N/A
Localization analysis: ProNPL::NPL-GFP//ProUBI::NLS-2xmCherry	This paper	N/A
Localization analysis: ProNPL::SyPME1-GFP//ProUBI::NLS-2xmCherry	This paper	N/A
Localization analysis, PME activity measurement: ProUBI::SyPME1-GFP//ProUBI::NLS-2xmCherry	This paper	N/A

(*Continued on next page*)

Continued

REAGENT or RESOURCE	SOURCE	IDENTIFIER
Phenotypical analysis, PME activity measurement: ProNPL::AtPMEI12-GFP//ProUBI:: NLS-2xmCherry	This paper	N/A
Phenotypical analysis: ProUBI::NPL-RNAi//ProUBI:: NLS-2xmCherry	This paper	N/A
Phenotypical analysis: ProLeEXT1::NPL-RNAi//ProUBI:: NLS-2xmCherry	This paper	N/A
Phenotypical analysis: ProAtPEP::NPL-RNAi//ProUBI:: NLS-2xmCherry	This paper	N/A
Promoter activity: ProLeEXT1::GUS//ProUBI:: NLS-2xmCherry	This paper and Goodchild and Bergersen [24]	N/A
Promoter activity: ProAtPEP::GUS//ProUBI::NLS-2xmCherry	This paper and Szczyglowski et al.[25] and Peaucelle et al.[26]	N/A
Empty vector: Dummy//ProUBI:: NLS-2xmCherry	This paper	N/A
Software and algorithms		
All box plots have been plotted using Graphpad Prism	Graphpad Prism 8	https://www.graphstats.net/
All statistical tests have been carried out using Graphpad Prism	Graphpad Prism 8	https://www.graphstats.net/
Adobe Photoshop (Figure editing and typesetting)	Photoshop CS6	https://www.adobe.com/products/photoshop.html
ZEN (blue version)	ZEN 3.0	https://www.zeiss.com/microscopy/int/products/microscope-software/zen.html
ImageJ	Schindelin et al. [38]	https://imagej.nih.gov/ij/
IMARIS	Imaris 7.2.3	https://imaris.oxinst.com/
Phylogenetic tree was built by iTOL	iTOL v6	https://itol.embl.de/

RESOURCE AVAILABILITY

Lead Contact

Further information and requests for resources and reagents should be directed to and will be fulfilled by the lead contact, Thomas Ott (Thomas.Ott@biologie.uni-freiburg.det).

Materials Availability

All constructs and antibodies used in this study are listed in the key resources table.

Data and code availability

This study did not generate any unique datasets and code. The published article includes all data generated or analyzed during this study.

EXPERIMENTAL MODEL AND SUBJECT DETAILS

The model plant used in this study is *Medicago truncatula*, its compatible rhizobium strain is *Sinorhizobium meliloti* 2011 (*S. m 2011*). *Agrobacterium rhizogenes* strain ARqua1 was used for hairy root transformations. Plants were grown in an environmentally-controlled growth chamber (24°C, 16/8 long-day cycle and a light intensity of 85 μmol*m-2*s-1).

METHOD DETAILS

Plant growth and hairy root transformation

Seeds of Medicago were washed 6 times with sterile tap water after being sterilized for 20 min with pure sulfuric acid (H_2SO_4). The seeds were then treated with bleach solution (12% NaOCl, 0.1% SDS) for 60 s and washed again 6 times with sterile tap water. The sterilized seeds were covered with sterile tap water for 2 hours (h) before being transferred to 1% agar plates and stratified at 4°C for 3 days in darkness. After stratification, seeds were kept in darkness at 24°C for 24 h for germination. The seed coat was removed from germinated seedlings, which were then used for hairy root transformation as previously described.[39] Transformed seedlings were first placed onto solid Fahräeus medium (containing 0.5 mM NH_4NO_3) and incubated in darkness (at 22°C) for three days, following 4 days at 22°C in white light with roots kept in the darkness. One week later, seedlings were transferred onto fresh Fahräeus medium (0.5 mM NH_4NO_3) for another 10 days. Afterwards, the transformed roots were screened and transformed plants were transferred to open pots for phenotyping.

Phenotyping

Both R108 and *SyPME1* mutant seedlings were directly grown in open pots (mixture of 1:1 quarzsand:vermiculite mixture, 2 plants/pot) after seed germination. Plants were watered with liquid Fahräeus medium (without nitrate, 30 mL/pot) and tap water (30 mL/pot) once a week, individually. The plants were then inoculated with *S. meliloti* (Sm2011; OD600 = 0.003) 7 days after transfer (20 mL/pot). Another 10 days later, plants were harvested for the quantification of infection structures.

Visualization of IT phenotypes

Roots transformed with the different NPL-RNAi constructs were harvested 10 days post-inoculation (dpi), fixed in PBS solution containing 4 % PFA under vacuum for 15 min (twice) and kept at room temperature for 2 h before being transferred to a ClearSee solution. Roots were kept in ClearSee for 2-3 days before the solution was refreshed and supplied with 0.1% Calcofluor white prior to imaging. For CLEM analysis, transformed roots harvested at 10 dpi and root fragments with infection events were first embedded in low melting agarose before fixation and subsequent processing as described below.

In situ hybridization

A 228 nucleotides fragment of the SyPME1 CDS was amplified using specific primers (key resources table) from *Medicago truncatula* A17 cDNA with KOD DNA polymerase (Stratagene, San Diego, CA, USA). The amplified fragment was cloned into a blunt cloning vector pEASY-Blunt3, which worked as the template for generating the Digoxigenin-labelled sense or antisense RNA probes using T7 RNA polymerase. Young nodule primordia (10 dpi) and matured nodules (20 dpi) were collected as material for further in situ hybridizations. Material preparation (fixation, dehydration, infiltration, and embedding) was performed as previously described earlier.[40] Paraffin-embedded samples were sectioned at a thickness of 10 μm for hybridization.

Cloning and DNA constructs

All initial DNA fragments being used in this study were synthesized by Life Technologies, before being assembled in Golden Gate compatible expression vectors.[41] All designed constructs and related primers used in this study are listed in key resources table. Golden Gate L0 modules for the ProLeEXT1[18] and ProAtPEP[19,20] promoters were kindly provided by Dr. Tatiana Vernié (University of Toulouse, France). All Medicago gene sequences were retrieved from the Phytozome database with the gene IDs: *SyPME1* (*Medtr4g087980*), *NPL* (*Medtr3g086320*).

GUS staining

GUS staining of roots and nodules was performed as described previously.[42] Stained nodules were embedded in Technovit 7100 for microtome sectioning and sections (10 μm) were further stained with 0.05% w/v Toluidine Blue prior to imaging. The sections from roots carrying the ProEXT1-GUS or the ProPEP-GUS were stained with 0.1% Ruthenium Red.

Immunofluorescence labelling

Nodule sections were blocked with 3% BSA for 30 min at room temperature before adding the primary antibody onto the sections (PlantProbes; 1:50 dilution in PBS solution supplemented with 3% BSA). Incubation with the primary antibody was carried out for 1 h at room temperature or overnight at 4°C in the case of 2F4. Samples were then washed at least three times for 5 min with PBS and the secondary antibody (conjugated with AlexaFluor 546 goat anti-rat or AlexaFluor 546 goat anti-mouse, Invitrogen; 1:500 dilution in PBS supplemented with 3% BSA) was added for an additional 30 min to one-hour incubation in darkness. Finally, the samples were washed at least three times with PBS in the dark prior to image acquisition. All the processes were performed on microscopy glass slides in a homemade humidity chamber at room temperature. When testing putative epitope masking, nodule sections were pre-treated with either xyloglucanase (XYLOGLUCANASE (GH5) (Paenibacillus sp.) (Megazyme, Lot 100702A)) or pectate lyase (PECTATE LYASE (Apergillus sp.) (Megazyme, Lot 170902C) to remove specific cell wall polysaccharides. Sample processing was performed as described earlier.[43]

For immunofluorescence quantification on WT and *npl* nodule sections, samples were embedded in Technovit 8100 for microtome sectioning (10-15 μm) and sections were further used for immunofluorescence labeling using antibodies LM19, LM20 and 2F4.

Fluorescence microscopy

Composite Medicago plants for fluorescence imaging were grown in open pots and inoculated with *S. meliloti* as described above. To study primary infection events, roots were harvested at 7 dpi while nodules were obtained at 2 wpi. Nodules were embedded in 7 % low melting agarose before 70 μm thick vibratome sections were obtained for subsequent confocal microscopy using a Leica TCS SP8 with the following settings: Images were acquired with a 20×/0.75 (HC PL APO CS2 IMM CORR) or a 40x/1.0 (HC PL APO CS2) water immersion objective (only for nodule vibratome sections). Genetically encoded fluorophores were excited using a White Light Laser (WLL) with GFP: 488 nm (ex) /500-550 nm (em); mCherry: 561 nm (ex) / 575-630 nm (em); Calcofluor white: 405 nm (ex; UV Laser) / 425-475 nm (em). Images for CLEM-related immunofluorescence were taken with a ZEISS ApoTome.2 light microscope with the following detection settings: CFP: BP 480/40 DMR 25; dsRed: BP 629/62. All the image analyses and projections were performed using either the ImageJ/(Fiji)[38] or Imaris software.

Sample preparation for TEM analysis

Nodules were sectioned in half and immediately fixed in MTSB buffer containing 8% paraformaldehyde (PFA) and 0,25 % glutaraldehyde (GA) at room temperature under vacuum for 15 min and left in the same fixative solution for 2 h at room temperature. After this, they were further fixed with 4% PFA and 0,125 % GA for 3 h at room temperature and with 2% PFA and 0,65 % GA overnight at 4°C. Nodules were then washed with MTSB buffer, and dehydrated in ethanol graded series at progressive low temperature as follows: 30% EtOH at 4°C 15 min, 50%-70%-95%-and 100% EtOH at -20°C for 15 min each, followed by a second incubation in 100% EtOH at -20°C for 30 min. Embedding in Lowicryl HM20 was performed at -20°C gradually increasing the EtOH/Lowicryl ratio (1:1 for 1h, 1:2 for 1h, 0:1 for 1h). After the last step, the resin was replaced with fresh Lowicryl and the samples were incubated overnight at -20°C. For polymerization, the resin was replaced once more, and the samples were kept at -20°C for 2 days under UV light. Blocks were sectioned with a Reichert-Jung Ultracut-E microtome. Ultrathin (70 nm) sections were collected in copper slot or finder grids and observed in a Philips CM10 (80 kV) microscope coupled to a GATAN Bioscan Camera Model 792 or a Hitachi 7800 TEM coupled to a Xarosa CMOS camera (Emsis).

Sample preparation for CLEM

CLEM was applied on 70 nm Lowicryl HM20 ultrathin sections collected on finder grids. The grids with the sections were washed with PBS buffer for 5 min, followed by an incubation with 0.12 M Glycine in PBS for 10 min. After 5 min washing in PBS, the grids were incubated for 10 min in blocking solution (3% BSA in PBS) followed by 30 min incubation with the first antibody in blocking solution. After six times washing for 3 min each in PBS, the grids were incubated for 30 min in blocking solution containing the above-mentioned fluorescence labelled secondary antibody. Grids were washed six times for 3 min each in PBS and incubated in 1% DAPI solution for 5 min before they were mounted on a microscope glass slide for observation with a fluorescence microscope.

Immuno-Gold staining

Samples for immuno-gold staining were treated as for CLEM but substituting the secondary antibody by conjugated Protein A-gold 5 nm (University Medical Center Utrecht) or Goat-anti Rat IgG 12 nm gold conjugated polyclonal antibody (Jackson Immuno Research (112-205-167)), and contrasting the sections with 2% uranyl acetate after washing in water.

Determination of total PME activities

PME activity was quantified by a radial gel diffusion experiment according as described earlier.[16] In brief, equal amounts of protein (10 μg) were loaded into wells (3 mm) on 1% (w/v) agarose gels containing 0.2% (w/v) 65% methylesterified apple pectin (Sigma-Aldrich), 12.5 mM citric acid, and 50 mM Na_2HPO_4, pH 6.5. Loaded gels were incubated at 28°C overnight before being stained with 0.005% ruthenium red (room temperature) for 2 h and de-stained with tap water. The stained areas were measured using Fiji.

QUANTIFICATION AND STATISTICAL ANALYSIS

Box-whiskers plots with individual dots (from Min to Max) were generated using GraphPad Prism 8. The box always extends from the 25th to the 75th percentiles. The line in the middle of the box is plotted at the median. Statistical significance was examined by two-tailed Student's t-test using GraphPad Prism 8. Sample sizes and statistical parameters are stated in the corresponding Figure legends. Fluorescence intensities were measured by using Fiji.

Current Biology, Volume *33*

Supplemental Information

Transcellular progression of infection threads in *Medicago truncatula* roots is associated with locally confined cell wall modifications

Chao Su, Guofeng Zhang, Marta Rodriguez-Franco, Rosula Hinnenberg, Jenny Wietschorke, Pengbo Liang, Wei Yang, Leonard Uhler, Xia Li, and Thomas Ott

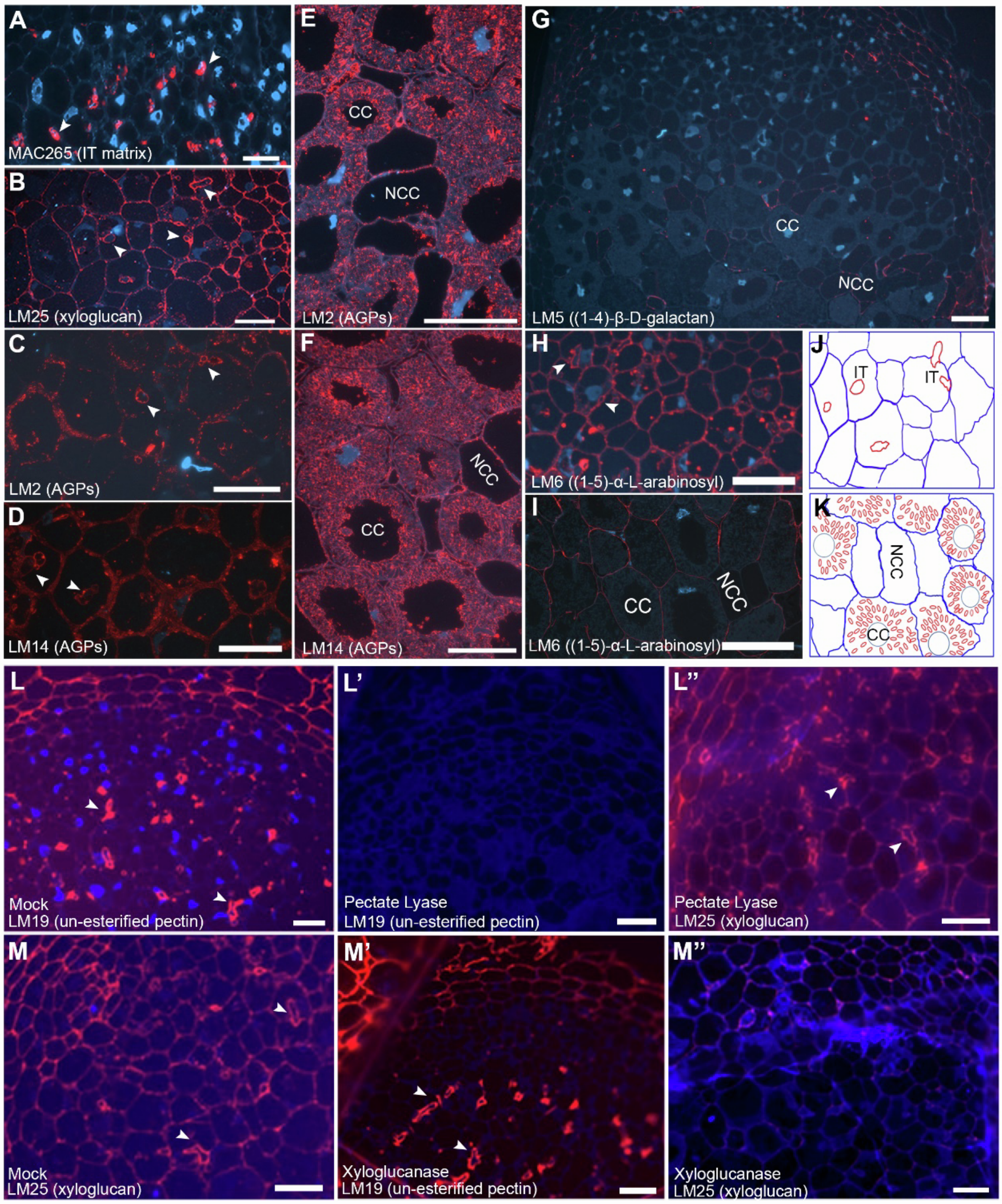

Figure S1: Immunofluorescence labeling with different cell wall antibodies, related to Figure 1. (A-I) 14 days old Medicago nodules were embedded in Lowicryl HM20 and hybridized with the respective antibodies (red). Cellular structures were labelled by different antibodies: (A) Infection thread (IT) matrix (MAC265); (B) xyloglucan (LM25); (C-F) arabinogalactan proteins (AGPs; LM2 (C, E), LM14 (D, F); different types of RG-I (LM5 (G) and LM6 (H, I)). Sections of nodule infection zone (A-D, H) and fixation zone (E, F, and I). (J-K) Illustrations indicate nodule sections from infection zone (J) and fixation zone (K). Red circles in (J) indicate ITs and symbiosomes in (K). Images in B, C, D and H as well as E and F were taken from consecutive sections from the same nodules but probed with different antibodies, respectively. (L-M''). Nodule sections were pre-treated with pectate lyase (L-L'') or xyloglucanase (M-M'') before being hybridized with different antibodies. DNA was counterstained with DAPI (blue). Images in L-M'' were taken from consecutive sections from the same nodule but probed with different antibodies. Arrowheads indicate infection threads. Scale bars indicate 50 µm. IT: infection thread, CC: colonized cell; NCC: non-colonized cell.

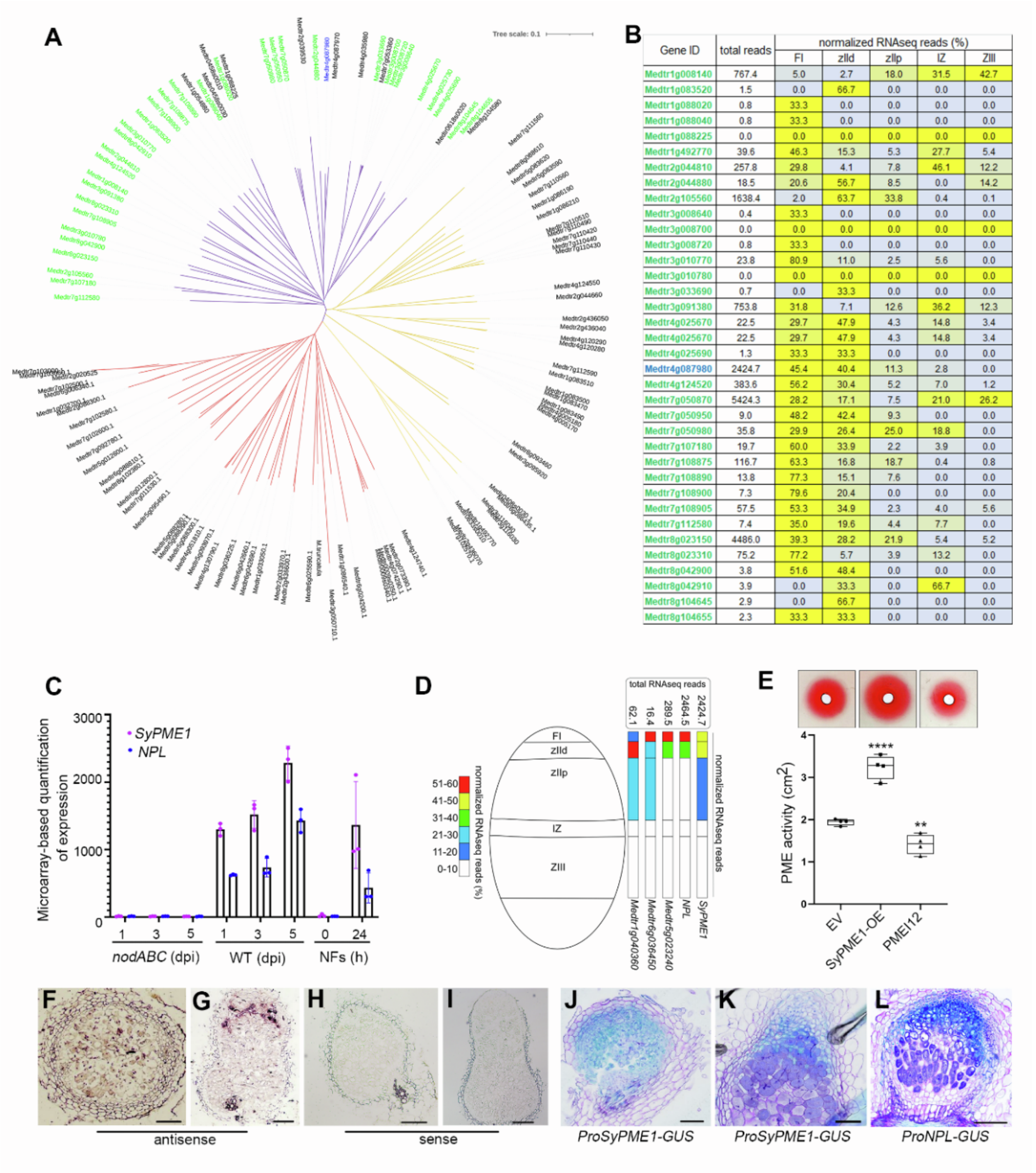

Gene ID	total reads	normalized RNAseq reads (%)				
		FI	zIId	zIIp	IZ	ZIII
Medtr1g008140	767.4	5.0	2.7	18.0	31.5	42.7
Medtr1g083520	1.5	0.0	66.7	0.0	0.0	0.0
Medtr1g088020	0.8	33.3	0.0	0.0	0.0	0.0
Medtr1g088040	0.8	33.3	0.0	0.0	0.0	0.0
Medtr1g088225	0.0	0.0	0.0	0.0	0.0	0.0
Medtr1g492770	39.6	46.3	15.3	5.3	27.7	5.4
Medtr2g044810	257.8	29.8	4.1	7.8	46.1	12.2
Medtr2g044880	18.5	20.6	56.7	8.5	0.0	14.2
Medtr2g105560	1638.4	2.0	63.7	33.8	0.4	0.1
Medtr3g008640	0.4	33.3	0.0	0.0	0.0	0.0
Medtr3g008700	0.0	0.0	0.0	0.0	0.0	0.0
Medtr3g008720	0.8	33.3	0.0	0.0	0.0	0.0
Medtr3g010770	23.8	80.9	11.0	2.5	5.6	0.0
Medtr3g010780	0.0	0.0	0.0	0.0	0.0	0.0
Medtr3g033690	0.7	0.0	33.3	0.0	0.0	0.0
Medtr3g091380	753.8	31.8	7.1	12.6	36.2	12.3
Medtr4g025670	22.5	29.7	47.9	4.3	14.8	3.4
Medtr4g025670	22.5	29.7	47.9	4.3	14.8	3.4
Medtr4g025690	1.3	33.3	33.3	0.0	0.0	0.0
Medtr4g087980	2424.7	45.4	40.4	11.3	2.8	0.0
Medtr4g124520	383.6	56.2	30.4	5.2	7.0	1.2
Medtr7g050870	5424.3	28.2	17.1	7.5	21.0	26.2
Medtr7g050950	9.0	48.2	42.4	9.3	0.0	0.0
Medtr7g050980	35.8	29.9	26.4	25.0	18.8	0.0
Medtr7g107180	19.7	60.0	33.9	2.2	3.9	0.0
Medtr7g108875	116.7	63.3	16.8	18.7	0.4	0.8
Medtr7g108890	13.8	77.3	15.1	7.6	0.0	0.0
Medtr7g108900	7.3	79.6	20.4	0.0	0.0	0.0
Medtr7g108905	57.5	53.3	34.9	2.3	4.0	5.6
Medtr7g112580	7.4	35.0	19.6	4.4	7.7	0.0
Medtr8g023150	4486.0	39.3	28.2	21.9	5.4	5.2
Medtr8g023310	75.2	77.2	5.7	3.9	13.2	0.0
Medtr8g042900	3.8	51.6	48.4	0.0	0.0	0.0
Medtr8g042910	3.9	0.0	33.3	0.0	66.7	0.0
Medtr8g104645	2.9	0.0	66.7	0.0	0.0	0.0
Medtr8g104655	2.3	33.3	33.3	0.0	0.0	0.0

Figure S2: Analysis of *SyPME* expression domains, related to Figures 2 and 3. (A) Maximum likelihood tree of the PME family in Medicago. All the (amino acid) sequences were obtained from *Phytozome v13* (https://phytozome-next.jgi.doe.gov/info/Mtruncatula_Mt4_0v1). The gene marked in blue is *SyPME1*. Genes in green letters fall into SyPME1 clade (A) and gene expression data for these genes were retrieved from [S1] (B). FI: nodule meristematic zone; zIId: distal of infection zone; zIIp: proximal of infection zone; IZ: interzone; ZIII: nitrogen-fixation zone. (C) Expression patterns of *NPL* and *SyPME1* are highly correlated (0.9883) as exemplified upon inoculation of roots with a Nod Factor (NF)-deficient *S. meliloti nodABC* strain (*nodABC*), WT *S. meliloti* and isolated NFs at different time points. Data were retrieved from [S2]. dpi: days post inoculation; h: hours. (D) Schematic overview of the spatial expression of SyPME and different pectate lyases including NPL in different zones of an indeterminate Medicago nodule. Original data were retrieved from [S1]. (E) Quantification analysis of PME activity from inoculated roots containing empty vector (EV), SyPME1-GFP (SyPME-OE), and PMEI12-GFP (PMEI12) (n = 4). Data are means ± SE. Statistics were performed using an unpaired two-tailed t-test: ****$p < 0.0001$; **$p < 0.01$. (F-I) Spatial analysis of *SyPME* transcript accumulations by i*n situ* hybridization using a *SyPME* antisense (F-G) and sense (control) probe (H-I) on 14-day old Medicago nodules. Magenta precipitates indicate presence of *SyPME* mRNA. (J-L) Promoter GUS (blue) analysis for *ProSyPME1* (J, K) and *ProNPL* (L) on 14-day old transformed *M. truncatula* nodules counterstained with Toluidine Blue (purple). Scale bars indicate 50 µm.

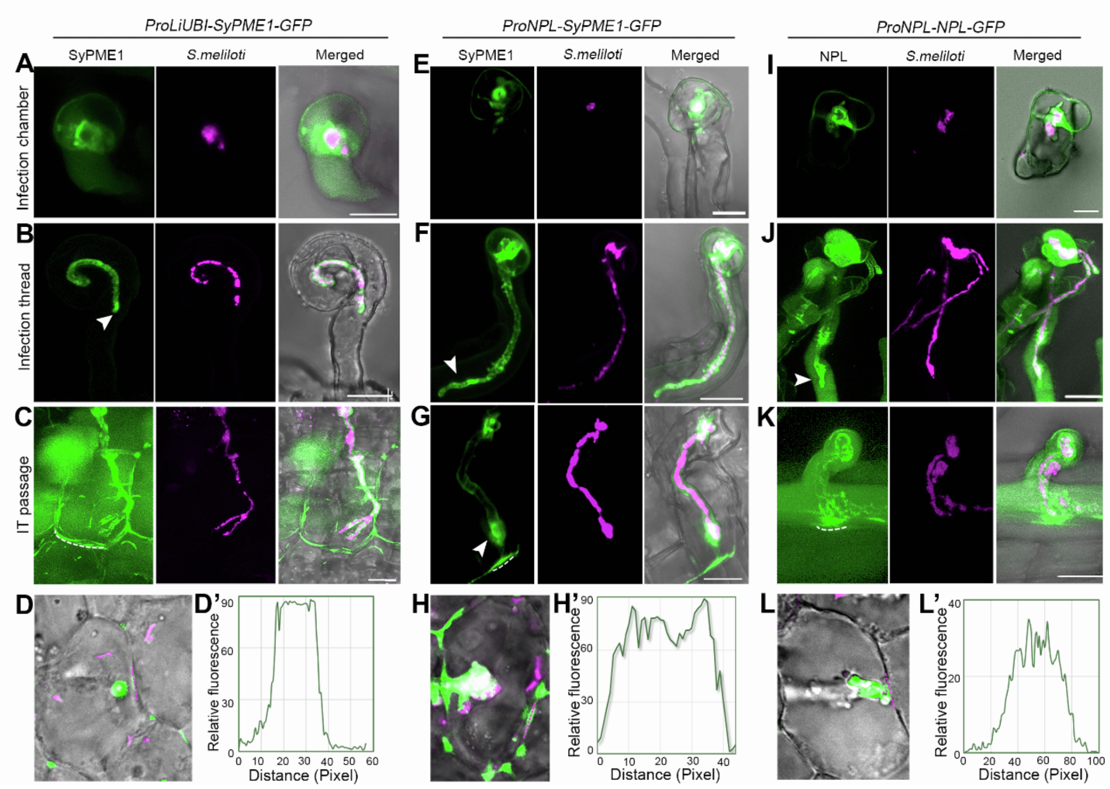

Figure S3: SyPME1 and NPL accumulate in spatially overlapping sites, related to Figure 2. Expression of a SyPME1-GFP fusion protein (green) was driven by the *ProLjUBI* promoter (A-D) or the *ProNPL* promoter (E-H). Expression of an NPL-GFP fusion protein (green) was driven by *ProNPL* (I-L). (A-B, E-F) SyPME1-GFP (green) localizes to the infection chamber (IC; A, E (n = 20)) and along growing infection threads (IT) including the IT tip (arrowhead, B, F (n = 15)). (I-J) Similar patterns were observed for NPL-GFP (green) at the IC (I, n = 20) while NPL predominantly localized to the IT tip (arrowhead) and young parts of the IT (J, n = 20). Initial labelling of future transcellular passage sites prior to IT passage was found for SyPME1 (C, G) and NPL (K) as indicated by the dashed line (n = 5 for C, n = 6 for G and n = 4 for K). Fluorescent *S. meliloti* is shown in magenta. All images are maximal projections. Scale bars indicate 10 µm. Inside nodule, SyPME1 (D, H) and NPL (L) also accumulate at prospective passage sites and at the tip of IT (images are identical with panels Figure 3B, H, N, respectively). Dashed magenta line indicates transect used to determine fluorescence intensities at these sites for SyPME1 (D', H') and NPL (L'). Data were collected based on at least three independent rounds of hairy root transformations.

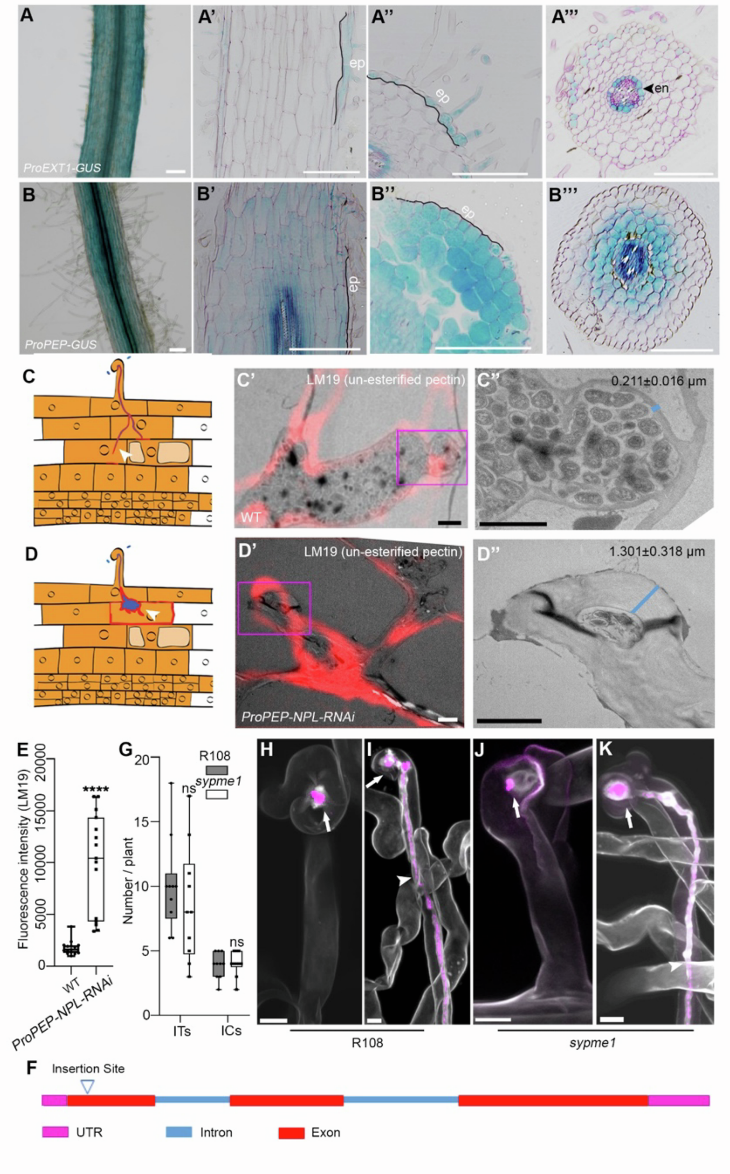

Figure S4: A single locus *sypme1* mutant does not exhibit a symbiotic phenotype, related to Figures 3 and 4. (A-B''') GUS staining of Medicago roots transformed with *ProEXT1-GUS* (A-A''') and *ProPEP-GUS* (B-B''') reporter constructs. Root longitudinal sections (A' and B') and cross sections (A''-A''', B''-B''') after GUS staining. ep: epidermis, en: endodermis. Scale bars indicate 100 μm. (C and D) Models illustrating the sites (arrowheads) that were used for CLEM analysis. (C-D'') CLEM analysis of sections of cortical cells from WT (C-C'', n=4) and *ProPEP-NPL-RNAi* transgenic roots (D-D'', n=3) penetrated by ITs and labelled with LM19 (red, C' and D'). (C'' and D'') Close-up of the boxed region (magenta) in C' and D', respectively. The blue bars in C'' and D'' indicate the thickness of IT cell walls and the numbers indicate means of 15 measurements from 3 independent samples. Scale bars indicate 2 μm. Quantitative immune-labelling of un-esterified pectins in (C' and D') is shown in E (n=15). Data are means ± SE. Statistics were performed using an unpaired two-tailed t-test: ****$p < 0.0001$. (F) Schematic representation of the *SyPME1* gene structure and mapped Tnt1 transposon insertion site (line NF2281). UTR, untranslated region. (G) Infection chambers (ICs) and infection threads (ITs) were scored at 10 days after inoculation, with n =10 root systems for each genotype. Data are means ± SE. Statistics were performed using an unpaired two-tailed t-test. ns: Not significant. (H-K) IC and IT morphology were visualized by Calcofluor-white staining (White color) in R108 (H-I) and *sypme1* mutants (J-K). Fluorescent *S. meliloti* is shown in magenta. Arrows indicate the IC and arrowheads indicate the IT. Scale bars indicate 10 μm. Images (H-K) are maximal projections.

SUPPLEMENTAL REFERENCES

S1. Roux, B., Rodde, N., Jardinaud, M.F., Timmers, T., Sauviac, L., Cottret, L., Carrere, S., Sallet, E., Courcelle, E., Moreau, S., et al. (2014). An integrated analysis of plant and bacterial gene expression in symbiotic root nodules using laser-capture microdissection coupled to RNA sequencing. Plant J *77*, 817-837. 10.1111/tpj.12442.

S2. Breakspear, A., Liu, C., Roy, S., Stacey, N., Rogers, C., Trick, M., Morieri, G., Mysore, K.S., Wen, J., Oldroyd, G.E., et al. (2014). The root hair "infectome" of Medicago truncatula uncovers changes in cell cycle genes and reveals a requirement for Auxin signaling in rhizobial infection. Plant Cell *26*, 4680-4701. 10.1105/tpc.114.133496.
